# Non-enzymatic hydrogen sulfide production from cysteine in blood is catalyzed by iron and vitamin B_6_

**DOI:** 10.1038/s42003-019-0431-5

**Published:** 2019-05-21

**Authors:** Jie Yang, Paul Minkler, David Grove, Rui Wang, Belinda Willard, Raed Dweik, Christopher Hine

**Affiliations:** 1 Department of Cardiovascular and Metabolic Sciences, Cleveland Clinic Lerner Research Institute, Cleveland, OH 44195 USA; 2 Proteomics and Metabolomics Core, Cleveland Clinic Lerner Research Institute, Cleveland, OH 44195 USA; 3 Department of Inflammation and Immunity, Cleveland Clinic Lerner Research Institute, Cleveland, OH 44195 USA; 4 Faculty of Science, Department of Biology, York University, Toronto, Canada M3J 1P3; 5 Respiratory Institute, Cleveland Clinic, Cleveland, OH 44195 USA

**Keywords:** Biochemistry, Physiology, Haematological diseases

## Abstract

Hydrogen sulfide (H_2_S) plays important roles in metabolism and health. Its enzymatic generation from sulfur-containing amino acids (SAAs) is well characterized. However, the existence of non-enzymatic H_2_S production from SAAs, the chemical mechanism, and its biological implications remain unclear. Here we present non-enzymatic H_2_S production in vitro and in blood via a reaction specific for the SAA cysteine serving as substrate and requires coordinated catalysis by Vitamin B_6_, pyridoxal(phosphate), and iron under physiological conditions. An initial cysteine-aldimine is formed by nucleophilic attack of the cysteine amino group to the pyridoxal(phosphate) aldehyde group. Free or heme-bound iron drives the formation of a cysteine-quinonoid, thiol group elimination, and hydrolysis of the desulfurated aldimine back to pyridoxal(phosphate). The reaction ultimately produces pyruvate, NH_3_, and H_2_S. This work highlights enzymatic production is inducible and robust in select tissues, whereas iron-catalyzed production contributes underappreciated basal H_2_S systemically with pathophysiological implications in hemolytic, iron overload, and hemorrhagic disorders.

## Abstract (web summary)

Jie Yang et al. show the non-enzymatic production of hydrogen sulfide from sulfur-containing amino acids (SAA) in vitro and in blood. They find that the reaction uses SAA cysteine as a substrate, and requires coordinated catalysis by Vitamin B6 and iron, all under physiological conditions.

## Introduction

Life on Earth has a long and storied history with the gas hydrogen sulfide (H_2_S). Before the advent of molecular oxygen-rich environments 2.3 billion years ago^1^, life depended on the utilization and consumption of H_2_S for many of its biochemical and cellular processes^2^. Paradoxically, several mass extinction events coincide with, and are hypothesized to be causative of, toxic H_2_S levels in the atmosphere and oceans^3^. Thus, life has evolved with H_2_S serving as both an essential nutrient and selective pressure, resulting in cellular pathways for its controlled production, utilization, and/or detoxification.

**Fig. 1:**
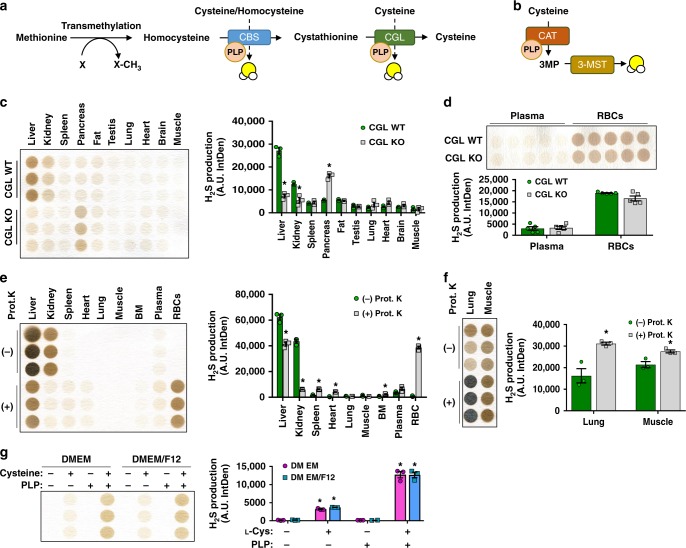
Enzymatic and non-enzymatic H_2_S production is tissue specific. **a**, **b** Generalized models of Vitamin B_6_ (VitB_6_) (PLP)-dependent enzymatic H_2_S production. H_2_S is generated from cysteine or homocysteine via the transsulfuration pathway enzymes, cystathionine β-synthase (CBS), and cystathionine γ-lyase (CGL) (**a**), or through the stepwise deamination of cysteine to 3-mercaptopyruvate (3-MP) by cysteine/asparatate aminotransferase (CAT) and release of H_2_S via 3-mercaptopyruvate sulfurtransferase (3-MST) (**b**). **c**, **d** H_2_S production from tissue extracts (**c**) (*n* = 3/group) or from plasma and red blood cells (RBCs) (**d**) (*n* = 5/group) collected from *CGL* WT and *CGL* KO mice in the presence of l-cysteine and PLP. Asterisk indicates the significance of the difference versus *CGL* WT; *P < 0.05. **e**, **f** H_2_S production capacity of tissue extracts, plasma, and RBCs from *CGL* WT mice in the presence of l-cysteine and PLP ± Proteinase K (Prot. K) pretreatment as measured after 3 h incubation (**e**) or 16 h incubation (**f**), *n* = 3/group. Asterisk indicates the significance of the difference versus sample without Prot. K pretreatment; *P < 0.05. **g** Non-enzymatic H_2_S production in DMEM media or DMEM/F12 media in the presence of cysteine and/or PLP; *n* = 3/group. Asterisk indicates the significance of the difference vs. media-only group; *P < 0.05. All data were presented as mean ± SEM

In mammals, enzymatically produced H_2_S and its associated hydrosulfide anion (HS^−^) and sulfide anion (S^2−^), herein collectively referred to as H_2_S^4^, are primarily derived from the metabolism of sulfur-containing amino acids (SAAs), specifically cysteine and homocysteine^5^. Three enzymes historically responsible for this production include cystathionine β-synthase (CBS) and cystathionine γ-lyase (CGL), which are part of the transsulfuration pathway (Fig. 1a), and 3-mercaptopyruvate sulfurtransferase (3-MST)^6,7^ (Fig. 1b). The enzymatic activities of CBS and CGL, while promiscuous in terms of substrates and yields^6^, require the pyridoxal phosphate (PLP) cofactor form of Vitamin B_6_ (VitB_6_) in α,β-elimination or β-replacement of the SAA thiol group to produce H_2_S^8,9^. However, although PLP is not required for 3-MST activity, it is required for its upstream enzyme cysteine aminotransferase (CAT) to degrade cysteine to 3-mercaptopyruvate, which serves as substrate for 3-MST to produce sulfane sulfur and eventual H_2_S^10^. Thus, enzymatic production of H_2_S from SAAs in mammalian tissues is dependent on PLP.

Deficiencies in endogenous enzymatic H_2_S production and levels are associated with detrimental health effects^11^. These include atherosclerosis^12^, hypertension^13^, and neurodegenerative disorders^14^. In addition, CGL deficiencies result in the failure to positively respond and adapt to dietary^15,16^ and ischemic^17^ preconditioning. Conversely, increasing endogenous H_2_S production capacity and/or levels provides healthspan and lifespan extending benefits in model organisms^11^. Thus, discovering new mechanisms that regulate and/or augment endogenous H_2_S pose novel scientific and therapeutic avenues.

Opposite of enzymatic synthesis pathways, endogenous production of H_2_S through non-enzymatic processes in mammalian tissues is not well understood or characterized. Sulfur-containing molecules found in garlic act as H_2_S donors when reduced with glutathione in red blood cells (RBCs)^18^ and thiosulfate serves as a source for H_2_S production under hypoxic conditions in select tissues^19^. However, whether SAAs serve as substrates for non-enzymatic H_2_S production in mammalian tissues under normal physiological conditions, the chemical mechanism(s) involved, and biological contexts are yet to be determined. In the current study, we investigate the status of non-enzymatic H_2_S production in various tissues and the chemical mechanism. Specifically, we uncover the biological relevance of VitB_6_, either in the pyridoxal or PLP form, and free- or hemin/heme-bound iron in catalyzing H_2_S from the SAA cysteine in vitro and in blood ex vivo using a variety of H_2_S- and metabolite-detecting approaches.

## Results

### Tissue-specific enzymatic and non-enzymatic H_2_S production

To establish enzymatic and non-enzymatic contributions to H_2_S production in various mammalian tissues, the protein expression of CBS, CGL, and 3-MST and H_2_S production capacities were determined. Expression of CBS and CGL were strongest in the liver and kidney, and 3-MST in the brain (Supplementary Fig. 1A,B). Other tissues, such as spleen, pancreas, abdominal white fat, testis, lung, heart, and skeletal muscle had relatively low expression of these three proteins (Supplementary Fig. 1A,B). As CGL contributes to the majority of enzymatic H_2_S production under physiological conditions, at least in the liver^20^, H_2_S production capacity was tested in *CGL* wild-type (WT) and knockout (KO) mice using the lead acetate/lead sulfide method^21^ with l-cysteine (L-Cys) as substrate and PLP as cofactor. H_2_S production was strongest in the liver and kidney from *CGL* WT mice, and *CGL* KO reduced production in these two tissues (Fig. 1c and Supplementary Fig. 1C). H_2_S production in other tissues, plasma, and RBCs, albeit low compared with that in the liver and kidney, were not decreased due to *CGL* deficiency (Fig. 1c, d).

We next tested whether CGL-independent H_2_S production is due to other H_2_S-producing enzymes or via a non-enzymatic mechanism. Pretreatment of tissues ex vivo with proteinase K (Prot. K) to remove enzymatic activity decreased H_2_S production in the liver and kidney (Fig. 1e and Supplementary Fig. 1D), whereas it unexpectedly increased H_2_S production in the spleen, heart, lung, muscle, bone marrow, and plasma, with the greatest increase in RBCs (Fig. 1e, f). Thus, hepatic and renal H_2_S production is predominantly enzymatic and driven by CGL, whereas non-enzymatic production is a major contributor in other tissues and in circulation.

Additional evidence for non-enzymatic H_2_S production was detected in vitro with cell culture media. Dulbecco’s modified Eagle’s medium (DMEM) + 10% serum alone produced H_2_S, albeit at a lower level compared with NCTC 1496 liver cells growing in DMEM + 10% serum, when spiked with L-Cys and PLP (Supplementary Fig. 1E). We next tested media without serum/plasma to serve as a catalyst for H_2_S production. L-Cys supplementation in addition to the cysteine/cystine basally present in media (Supplementary Table 1) DMEM and DMEM/F12 produced H_2_S, which was further enhanced with additional PLP (Fig. 1g and Supplementary Fig. 1F). Thus, PLP enhanced non-enzymatic H_2_S production from L-Cys in multiple tissues and in cell culture media. However, the identity of the catalytic factor(s) besides pyridoxine(phosphate) in the media and tissues that gives rise to non-enzymatic H_2_S production is yet to be identified.

### Fe^3+^ and PLP coordinately catalyze H_2_S production from L-Cys

**Fig. 2:**
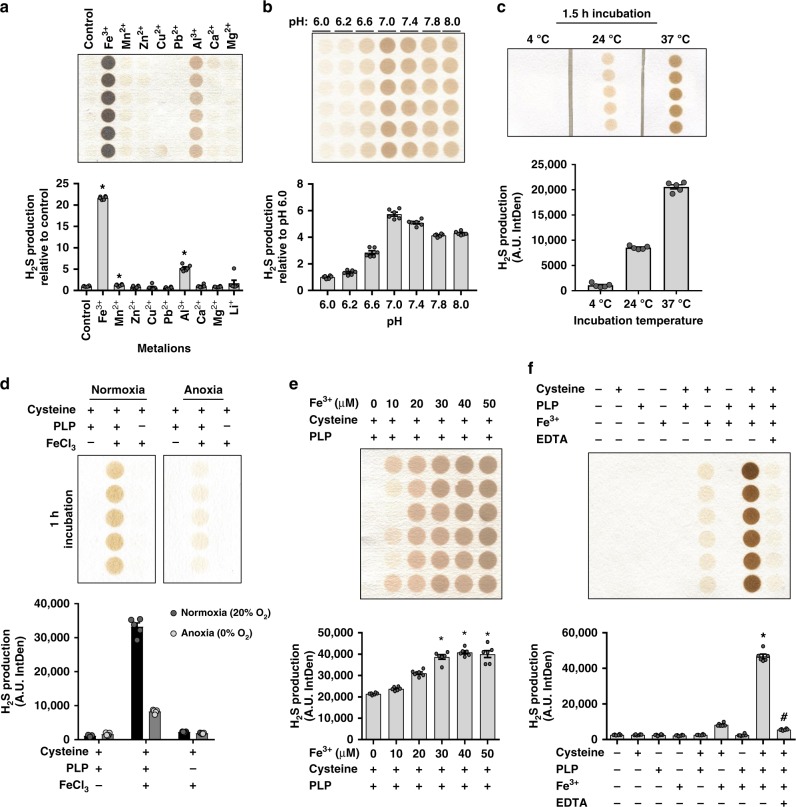
Fe^3+^ and PLP coordinate to catalyze H_2_S production from l-cysteine under physiological conditions. **a** H_2_S production catalyzed by inorganic metal ions as indicated, in the reaction mixture of l-cysteine and PLP; *n* = 6/group. Asterisk indicates the significance of the difference versus the l-cysteine and PLP alone reaction mixture control group; *P < 0.05. **b**–**e** The effect of pH (**b**; *n* = 6/group), temperature (**c**; *n* = 5/group), O_2_ (**d**; *n* = 5/group), and Fe^3+^ concentration (**e**; *n* = 6/group) on H_2_S production from reaction mixtures containing l-cysteine, PLP, and Fe^3+^ (**b**, **c**, **e**) or the l-cysteine and PLP mixture group (**d**); *P < 0.05. **f** H_2_S production in PBS ± l-cysteine ± PLP, ±Fe^3+^, and with the pretreatment of EDTA; *n* = 6/group. Asterisk indicates the significance of the difference vs. the l-cysteine and PLP mixture group, and pound indicates the significance of the difference between PBS ± l-cysteine ± PLP, ±Fe^3+^ with and without EDTA; *,^#^*P* < 0.05. All data were presented as mean ± SEM

Metal ions serve enzymatic and non-enzymatic catalytic roles^22^. Metal ion formulations for DMEM and DMEM/F12 (Supplementary Table 1) include iron (Fe^3+^), zinc (Zn^2+^), copper (Cu^2+^), and magnesium (Mg^2+^). These same metals are found in milligram to gram amounts in the human body, with iron and copper previously indicated to catalyze H_2_S and/or sulfide production from SAAs in coordination with pyridoxal under non-physiological conditions of temperature and/or pH^23,24^. We hypothesized that one or more of these metal ions catalyze non-enzymatic H_2_S production under physiological conditions. Ethylenediaminetetraacetic acid (EDTA), a metal ion chelator, inhibited H_2_S production in DMEM/F12 media (Supplementary Fig. 2). We next identified metal ions that act as catalysts for PLP-dependent H_2_S production from L-Cys in phosphate-buffered saline (PBS) at pH 7.4 and 37 °C (Fig. 2a). Iron (Fe^3+^) showed the greatest catalytic ability, followed by aluminum (Al^3+^) and, to a lesser extent, manganese (Mn^2+^). The other metal ions, Zn^2+^, Cu^2+^, Pb^2+^, Ca^2+^, and Mg^2+^, did not stimulate H_2_S production (Fig. 2a).

We next focused on iron, as it is abundant in the body, had the greatest catalytic activity (Fig. 2a), and is predominantly found in RBCs, which had the greatest increase in non-enzymatic H_2_S production after Prot. K treatment (Fig. 1e). Fe^3+^-induced production was optimum at a physiological pH 7.0–7.4 (Fig. 2b), temperature of 37 °C (Fig. 2c), normoxic 20% oxygen conditions (Fig. 2d), and was dose dependent in the µM range (Fig. 2e). Although Fe^3+^ somewhat catalyzed H_2_S from L-Cys without PLP present, possibly due to direct thiol oxidation and/or an alternate chemical mechanism, the addition of PLP greatly increased the production capacity (Fig. 2f). This indicates PLP serves as a vital cofactor for the formation of H_2_S from enzymatic and non-enzymatic Fe^3+^-mediated catalysis. EDTA chelation of Fe^3+^ dampened H_2_S production in PBS (Fig. 2f) and DMEM/F12 (Supplementary Fig. 2). Taken together, H_2_S is produced via the coordinated catalysis of L-Cys by Fe^3+^ and PLP under physiological conditions of pH, temperature, and oxygen.

### Gaseous and aqueous detection of Fe^3+^ and PLP-catalyzed H_2_S

**Fig. 3:**
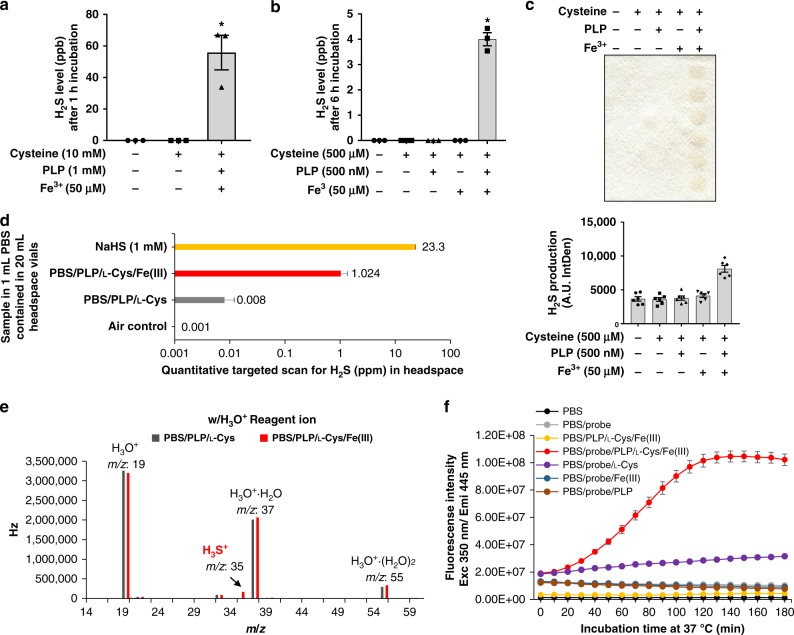
Detection of headspace and dissolved H_2_S catalyzed by Fe^3+^ and PLP with l-cysteine as substrate. **a**, **b** H_2_S levels (parts per billion; p.p.b.) in 6 mL headspace vials detected using the Jerome J605 after (**a**) 1 h incubation of reaction mixture containing supraphysiological concentration of l-cysteine (10 mM), PLP (1 mM), and Fe^3+^ (50 µM); *n* = 3/group, or after (**b**) 6 h incubation of reaction mixture containing more relevant physiological concentrations of l-cysteine (500 µM), PLP (500 nM), and Fe^3+^ (50 µM); *n* = 3/group. Asterisk indicates the significance of the difference versus the PBS background control; *P < 0.05. **c** Lead acetate/lead sulfide H_2_S production analysis under similar physiological conditions as in **b** with overnight exposure at 37 °C; *n* = 6/group. **d**, **e** Headspace H_2_S detected in the selected ion flow tube mass spectrometry (SIFT-MS) with **d** quantitative targeted scan for H_2_S; *n* = 1 for air control and NaHS groups, and *n* = 2 for PBS/PLP/L-Cys and PBS/PLP/L-Cys/Fe(III) groups, and **e** truncated mass spectrum over the range of mass-to-charge (*m*/*z*) shown in H_3_O^+^ reagent ion, measuring the product ions generated in the reaction with H_3_O^+^, being the *m*/*z* 35 product ion. The precursor ion signals (H_3_O^+^·(H_2_O)_0,1,2_ as appropriate) and the product ion signals are indicated. The concentrations of the trace gases are given in parts per million (p.p.m.). For full mass spectrum over the *m*/*z*, please see Supplementary Fig. 3c, d. **f** Time-dependent detection of dissolved H_2_S using the fluorogenic AzMC probe (Exc 350 nm/Emi 445 nm) from various reaction mixtures; *n* = 6/group. All data were presented as mean ± SEM

Additional sensitive and selective headspace or in-solution H_2_S detection techniques were used to confirm and expand upon our lead acetate/lead sulfide results presented in Fig. 1 and Fig. 2. The Jerome J605, a gold film H_2_S analyzer capable of measuring H_2_S in the 3 parts per billion (p.p.b.) to 1 parts per million (p.p.m.) range^25^ (Supplementary Fig. 3A), was configured to detect H_2_S produced from 1 mL reaction mixtures in 6 mL headspace vials (Fig. 3a, b and Supplementary Fig. 3B). Incubation of the complete reaction mixture containing supraphysiological levels of L-Cys, PLP, and Fe^3+^ in PBS for 1 h at 37 °C produced detectable H_2_S between 40 and 65 p.p.b. (Fig. 3a). H_2_S was not detected in the two control reaction solutions containing only PBS with or without L-Cys (Fig. 3a). Incubation of physiologically relevant amounts of L-Cys, PLP, and Fe^3+^ in PBS for 6 h produced detectable H_2_S between 3.5 and 4.5 p.p.b. (Fig. 3b). However, reaction mixtures not containing this full complement of substrate and catalysts produced no detectable H_2_S. We further confirmed H_2_S production from these physiologically relevant concentrations using the lead acetate/lead sulfide method with a relatively long 24 h incubation/exposure time at 37 °C (Fig. 3c).

We next employed selected ion flow tube mass spectrometry (SIFT-MS)^26,27^ as a quantitative, selective, and sensitive third headspace analysis method. Incubation of the complete reaction mixture containing L-Cys, PLP, and Fe^3+^ in PBS in a sealed 20 mL headspace vial for 30 min produced 1.024 p.p.m. H_2_S (Fig. 3d). H_2_S from control reaction mixtures lacking Fe^3+^ and in the control room air was detected at 0.008 and 0.001 p.p.m., respectively. Comprehensive volatile molecule analysis and full spectrum over the range of mass-to-charge (*m*/*z*) in H_3_O^+^ reagent ion mode^27,28^ was used for samples with reaction mixtures of PLP, L-Cys, ±Fe^3+^ to detect all potential sulfide products in the headspace. H_2_S, being the *m*/*z* 35 product ion H_3_S^+^^27,28^, was the sole product detected in the full reaction mixture not present in the control lacking Fe^3+^ (Fig. 3e and Supplementary Fig. 3C,D). No additional smaller or larger mass sulfide species were detected (Supplementary Fig. 3C,D), confirming H_2_S as the major sulfide product.

H_2_S exists not just in the gas phase but is soluble in aqueous and organic solutions as well. We next determined the presence of non-enzymatically produced H_2_S in solution using the sulfide-sensitive 7-azido-4-methylcoumarin (AzMC) fluorogenic probe, which increases in florescence intensity at *λ*_Ex/Em_ = 350/445 nm upon H_2_S-mediated reduction^29,30^. Time-dependent increased florescence intensity was detected only with the full reaction mixture containing PBS, L-Cys, PLP, and Fe^3+^ (Fig. 3f). Thus, H_2_S produced from iron- and PLP-coordinated catalysis of L-Cys is detectable as a gas and in solution.

### Cysteine as optimum substrate for Fe^3+^- and PLP-catalyzed H_2_S

**Fig. 4:**
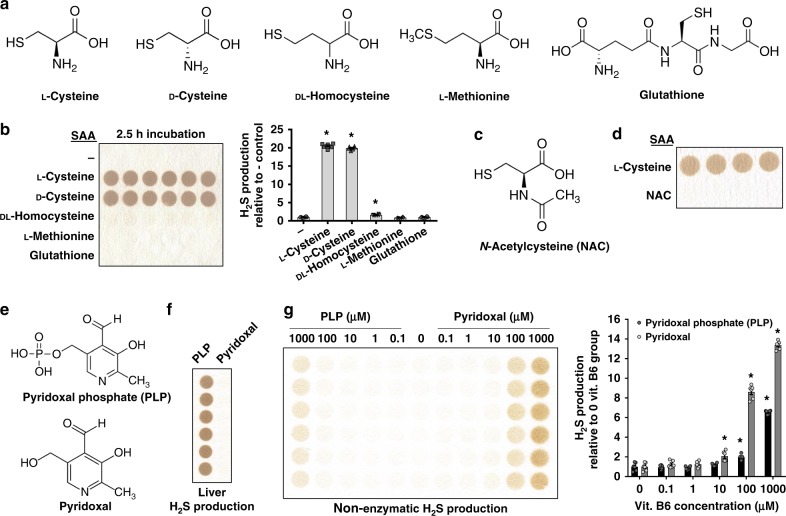
l- and d-Cysteine, but not other SAAs, act as substrate for rapid H_2_S production catalyzed by Fe^3+^ and PLP. **a** Chemical structure of the sulfur amino acids (SAAs). **b** H_2_S production capacity as determined by the lead acetate/lead sulfide method after 2.5 h incubation with different SAAs as substrate catalyzed by Fe^3+^ and PLP; *n* = 6/group. Asterisk indicates the significance of the difference versus the l-cysteine and PLP-alone control mixture group; *P < 0.05. **c** Chemical structure of *N*-acetylcysteine (NAC). **d** H_2_S production capacity with NAC as substrate in the presence of Fe^3+^ and PLP; *n* = 4/group. **e** Chemical structures of PLP and pyridoxal. **f**, **g** Selectivity of PLP and pyridoxal as co-factors for enzymatic H_2_S production in liver extract (**f**); *n* = 6/group, and non-enzymatic iron-catalyzed H_2_S production (**g**); *n* = 6/group. Asterisk indicates the significance of the difference versus the control PLP/Pyridoxal-null reaction mixture 0; *P < 0.05. Data were presented as mean ± SEM

Enzymatic H_2_S production utilizes SAAs as substrate, mainly L-Cys and homocysteine^6^. Although we have shown L-Cys is a substrate for Fe^3+^/PLP-driven non-enzymatic H_2_S production, it is not known whether other SAAs serve as substrates. We tested SAAs L-Cys, D-Cys, dl-Homocysteine, l-Methionine, and the tripeptide Glutathione (Fig. 4a) for production of H_2_S via PLP and Fe^3+^ catalysis. H_2_S production was specific for L- and D-Cys, and was detected after 2.5 h incubation (Fig. 4b). This is in contrast to enzymatic production via the liver, in which L-Cys, but not D-Cys, serves as a substrate (Supplementary Fig. 4A). Thus, the isomeric form, although important for enzymatic production, does not impact non-enzymatic production. Homocysteine did not provide the same kinetics as cysteine, as detectable H_2_S production by dl-Homocysteine was only apparent after a 16 h incubation (Fig. 4b and Supplementary Fig. 4B). This was not due to the DL-isomeric form, as DL- and L-Homocysteine performed similarly at 2 and 24 h incubations (Supplementary Fig. 4C). Acetylation of the amino group of cysteine, termed *N*-acetylcysteine (NAC) (Fig. 4c), blocked non-enzymatic H_2_S production (Fig. 4d). Cystine (Supplementary Fig. 4D), the oxidized dimer form of cysteine, failed to serve as substrate for H_2_S production (Supplementary Fig. 4E). Thus, cysteine in the L- or D-form serves as the best SAA substrate for H_2_S production via Fe^3+^- and PLP-coordinated catalysis, and this requires access to its amino group.

Besides the above SAAs, garlic-derived polysulfides are reduced by glutathione to produce H_2_S in human RBCs^18^. We tested whether they serve as substrates for Fe^3+^- and PLP-catalyzed H_2_S production. Two major volatile sulfur-containing garlic components, diallyl disulfide (DADS) and diallyl trisulfide (DATS) (Supplementary Fig. 4F), in addition to raw garlic blend, did not serve as compounds for H_2_S detection via the lead acetate/lead sulfide method when incubated solely in PBS or substrates for H_2_S production when incubated with Fe^3+^ and PLP (Supplementary Fig. 4G). In addition, the volatile sulfur-containing reducing reagents, dithiothreitol (DTT) and 2-mercaptoethanol (2ME) (Supplementary Fig. 4H), did not serve as compounds for H_2_S detection when incubated solely in PBS or substrates for H_2_S production when incubated with Fe^3+^ and PLP (Supplementary Fig. 4I, J). However, DTT reacted with Fe^3+^ independent of PLP and released H_2_S (Supplementary Fig. 4J), thus delivering H_2_S in a mechanism distinct from the reaction with cysteine. Importantly, the failure of these volatile organic sulfur compounds to form brown spots (lead sulfide) on the lead acetate paper independently of any chemical reaction supports the selectivity of the lead acetate/lead sulfide detection method for H_2_S and less so for other volatile sulfur-containing compounds.

The bioactive phosphorylated form of VitB_6_, PLP (Fig. 4e), is required as a cofactor for a variety of enzymatic reactions, including H_2_S biogenesis^10^. However, it is not known whether the phosphorylated form is required for non-enzymatic production of H_2_S with L-Cys as substrate. The majority of VitB_6_ in the body does not exists in the PLP form, but in the pyridoxal form^31^ (Fig. 4e). Thus, if non-enzymatic H_2_S production occurs with PLP and pyridoxal, it signifies this reaction is potentially more prevalent in vivo. We tested PLP and pyridoxal for both enzymatic (Fig. 4f) and non-enzymatic (Fig. 4g) H_2_S production. Liver-derived enzymatic H_2_S production required only PLP (Fig. 4f). Conversely, Fe^3+^-catalyzed non-enzymatic production of H_2_S occurred similarly with PLP and pyridoxal, signifying the phosphate group as dispensable (Fig. 4g). This, in combination with the requirement for access to the amino group of cysteine, gives clues to the chemical reaction mechanism of iron- and VitB_6_-coordinated production of H_2_S.

### Multi-step mechanism for Fe^3+^- and VitB_6_-catalyzed H_2_S

**Fig. 5:**
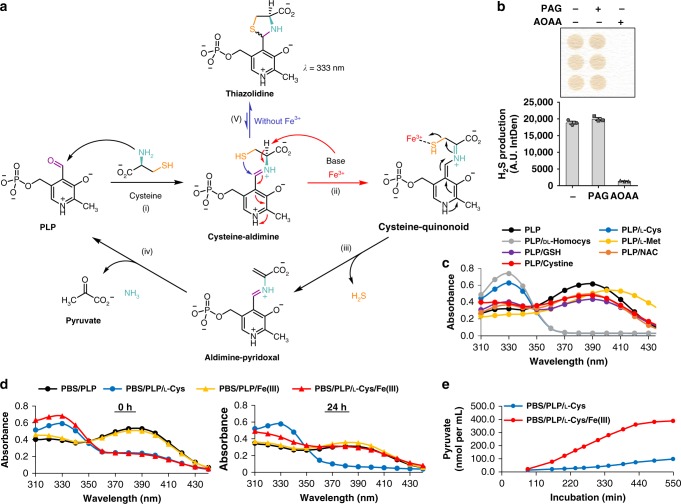
Mechanistic model for Fe^3+^- and PLP-catalyzed H_2_S production. **a** Proposed reaction model for H_2_S production from cysteine, PLP, and Fe^3+^: (i) the nucleophilic attack by the free amino group of cysteine on PLP forms a Schiff base, the cysteine-aldimine; (ii) deprotonation at α-position of cysteine leads to the formation of a quinonoid intermediate; (iii) the elimination of ‒SH group catalyzed by Fe^3+^; and (iv) the desulfurated aldimine is hydrolyzed to produce pyruvate, NH_3_, and regenerate PLP; (v) in the absence of Fe^3+^, a thiazolidine ring is formed from cysteine-aldimine product from step (i), offering UV/VIS peak absorbance at 333 nm^37^. **b** AOAA, but not PAG, inhibits Fe^3+^- and PLP-catalyzed H_2_S production; *n* = 3/group. Asterisk indicates the significance of the difference vs. the l-cysteine, Fe^3+^, and PLP control reaction mixture group; *P < 0.05. **c** Detection of PLP Schiff base by UV/VIS spectrophotometer. Absorbance of PLP in the absence or presence of tested sulfur amino acids was measured in the spectrum of 310–435 nm. The formation of Schiff base at ~330 nm is indicated by the shift of the peak with the addition of tested sulfur amino acids from ~390 nm. **d** Changes in absorbance in reaction mixture of PLP ± cysteine ± Fe^3+^ with reaction time. Loss of absorbance at 330 nm and gain at 390 nm after 24 h incubation in reaction mixture of cysteine with Fe^3+^ and PLP indicate Fe^3+^ consumes the substrate of cysteine and PLP is regenerated. **e** Pyruvate formation in reaction mixture of cysteine and PLP, ±FeCl_3_; *n* = 4/group. Data are measured colorimetrically at absorbance of 570 nm showing quantity (nmol) per ml H_2_S reaction mixture. All data were presented as mean ± SEM

Based on the intermolecular interactions between PLP with cysteine^10^ and serine^32^ in the context of enzyme-catalyzed elimination reactions, we hypothesized the enzyme-free chemical reaction to produce H_2_S consists of five generalized steps (Fig. 5a): (i) nucleophilic attack by the free amino group of cysteine on the aldehyde group of PLP or pyridoxal forms a Schiff base and subsequent cysteine-aldimine; (ii) deprotonation at the α-position of cysteine to form a quinonoid intermediate stabilized via the electron sink properties of PLP; (iii) Fe^3+^-catalyzed elimination of the thiol group forms a desulfurated aldimine and releases H_2_S; (iv) removal and hydrolyzation of the desulfurated cysteine to produce pyruvate, ammonia (NH_3_), and regenerate PLP (or pyridoxal), and (v) in the absence of Fe^3+^, a five-member thiazolidine ring is made from the cysteine-aldimine product from step (i). In the following section, we present empirical data along with published studies supporting the proposed chemical mechanism.

In (i), the amino group of cysteine attacks the aldehyde group of PLP to form the Schiff base and subsequent cysteine-aldimine. Blocking this interaction via acylation of the amino group on cysteine inhibited the production of H_2_S (Fig. 4d). Similarly, aminooxyacetic acid (AOAA) (Supplementary Fig. 5A), a proposed inhibitor of PLP-dependent enzymes and of enzymatic H_2_S production^33^ (Supplementary Fig. 5B) due to it outcompeting the enzyme to form a Schiff base linkage with the aldehyde group of PLP^30,34^, inhibited non-enzymatic H_2_S production (Fig. 5b). However, propargylglycine (PAG) (Supplementary Fig. 5A), an inhibitor of CGL (Supplementary Fig. 5B) due to it covalently modifying the enzyme vs. PLP itself^30,33^, had no effect on non-enzymatic H_2_S production (Fig. 5b).

We further confirmed the requirements for the formation of the cysteine-aldimine via UV/Spec absorbance analysis of the PLP-derived Schiff base with SAAs and AOAA by examining the shift in *λ*_Max_, which is ~388 nm for free PLP at physiological pH, to a peak in the 320–330 nm range for the Schiff base^35^ (Fig. 5c, Supplementary Fig. 5C,D,E, and Table 1). As a control, the SAAs alone did not display a distinctive *λ*_Max_ in this region (Supplementary Fig. 5C). PLP with AOAA resulted in a complete loss of the 388 nm peak and a shift to 324 nm (Supplementary Fig. 5D). PLP with L-Cys or dl-homocysteine, the two SAAs that produced H_2_S albeit with different kinetics, displayed a loss of *λ*_Max_ 388 nm and a shift to the 320–330 nm peak (Fig. 5c and Supplementary Fig. 5D). SAAs that failed to serve as substrate for H_2_S production (Fig. 4b, d and Supplementary Fig. 4E) did not remove the *λ*_Max_ at 388 nm and formed no peaks or incomplete peaks in the 320–330 nm range (Fig. 5c, Supplementary Fig. 5E, and Table 1). Notably, in the absence of Fe^3+^, a thiazolidine ring can be formed from the cysteine-aldimine product in step (i) by intramolecular attack of the sulfhydryl group to the imine carbon atom^36^, which also has a *λ*_Max_ in the 320–330 nm range^37^. Therefore, the *λ*_Max_ in the 320–330 nm range detected in the current study indicates cysteine-aldimine and/or thiazolidine, and the presence of iron prevents and/or reopens the thiazolidine ring for downstream sulfide removal. The role for Fe^3+^ catalytically consuming cysteine and subsequent regeneration of the PLP cofactor was also investigated via UV/Spec analysis (Fig. 5d). Addition of L-Cys to PLP immediately shifted the *λ*_Max_ from ~388 nm to 320–330 nm, indicative of the Schiff base/thiazolidine ring (Fig. 5d). Further addition of Fe^3+^ to this reaction mixture resulted in loss of *λ*_Max_ at 320–330 nm and gain at ~388 nm after a 24 h incubation at 37 °C (Fig. 5d).

**Table 1:** UV/Vis spectrophotometer-based detection of Schiff base formation between PLP and sulfur-containing amino acids

Reactants	1st Peak absorbance wavelength (nm)	*λ*_Max_ OD at peak 1	2nd Peak absorbance wavelength (nm)	λ_Max_ OD at peak 2
PLP	386–388	0.62	ND	ND
*PLP* *+* *L-Cys*	330	0.63	ND	ND
*PLP* *+* *DL-Hmcys*	328–330	0.74	ND	ND
PLP + L-Met	404	0.54	ND	ND
PLP + GSH	328–330	0.42	384–396	0.4
PLP + NAC	328	0.38	386–388	0.49
PLP + Cystine	390	0.52	ND	ND

The sulfur-containing amino acids selected as substrate for non-enzymatic H_2_S production are in italic. The numerical values presented in this table correspond to the plotted values in Fig. 5c and Supplementary Fig. 5c–e. *DL-Hmcys* dl-homocysteine, *GSH* glutathione, *L-Cys* l-cysteine, *L-Met* l-methionine, *NAC* *N*-acetylcysteine, *ND* not detected, *PLP* pyridoxal phosphate

We next utilized a liquid chromatography-tandem MS (LC-MS/MS) metabolomics platform to identify intermediaries and final products of the proposed reaction. Combinations of L-Cys, PLP, and Fe^3+^ were incubated in PBS at 37 °C before analysis. L-Cys with PLP resulted in the identification of the cysteine-aldimine/cysteine-quinonoid (Table 2 and Supplementary Fig. 5H). All of the detectable intermediates and products, including aldimine and pyruvate, were only found with the addition of Fe^3+^ (Table 2 and Supplementary Fig. 5I,J,M). In addition, Fe^3+^ lowered the peak heights for cysteine and cysteine-aldimine/cysteine-quinonoid, indicating Fe^3+^ is required to catalyze the reaction beyond the cysteine-aldimine/thiazolidine step (Table 2 and Supplementary Fig. 5I,J). As Fe^3+^-catalyzed H_2_S production is enhanced at 37 °C (Fig. 2c), we examined the peak heights of the full reaction incubated at 37 °C vs. 4 °C to determine which steps are temperature dependent. Peak heights were lower for cysteine and cysteine-aldimine/cysteine-quinonoid and higher for pyruvate at 37 °C compared with 4 °C (Table 2 and Supplementary Fig. 5I-M). However, the formation of the cysteine-aldimine/cysteine-quinonoid still occurred at 4 °C (Table 2 and Supplementary Fig. 5L). Time-dependent pyruvate formation was additionally confirmed using a colorimetric-based assay (Fig. 5e). Fe^3+^ enhanced the rate of pyruvate formation compared with the reaction mixture lacking Fe^3+^ (Fig. 5e). Taken together, the initial step of Schiff base/thiazoladine and cysteine-aldimine/cysteine-quinonoid formation between cysteine and VitB_6_ is not temperature dependent and does not require Fe^3+^, but does require free access to both the aldehyde group on VitB_6_ and the amino group of cysteine. However, subsequent steps in the reaction require Fe^3+^ and physiological temperatures to efficiently proceed and ultimately produce H_2_S, pyruvate, NH_3_, and recycle VitB_6_.

**Table 2:** Expected intermediates and products identified and their relative yields as determined from LC-MS/MS full scan extracted ion chromatograms

Reactants	l-Cysteine	Cysteine-aldimine/cysteine-quinonoid	Aldimine	Pyruvate
LC-MS/MS products identified				
L-Cys	+	ND	ND	ND
L-Cys + PLP	+	+	ND	ND
*L-Cys* *+* *PLP* *+* *Fe*^3+^	+	+	+	+
LC-MS/MS chromatographic peak heights				
L-Cys	274,723	ND	ND	ND
L-Cys + PLP	270,622	127,0041	ND	ND
* L-Cys* + *PLP* + *Fe*^3+^ (*37* °C)	6868	43,177	34,500	25,200
L-Cys + PLP + Fe^3+^ (4 °C)	10,387	338,581	32,500	10,450

Italic text indicates reactions and conditions favorable for H_2_S production. The data and numerical values presented in this table correspond to the plotted values in Supplementary Fig. 5F-M. *L-Cys* l-cysteine, *ND* not detected, *PLP* pyridoxal phosphate

### Non-enzymatic produced H_2_S in blood requires iron and VitB_6_

**Fig. 6:**
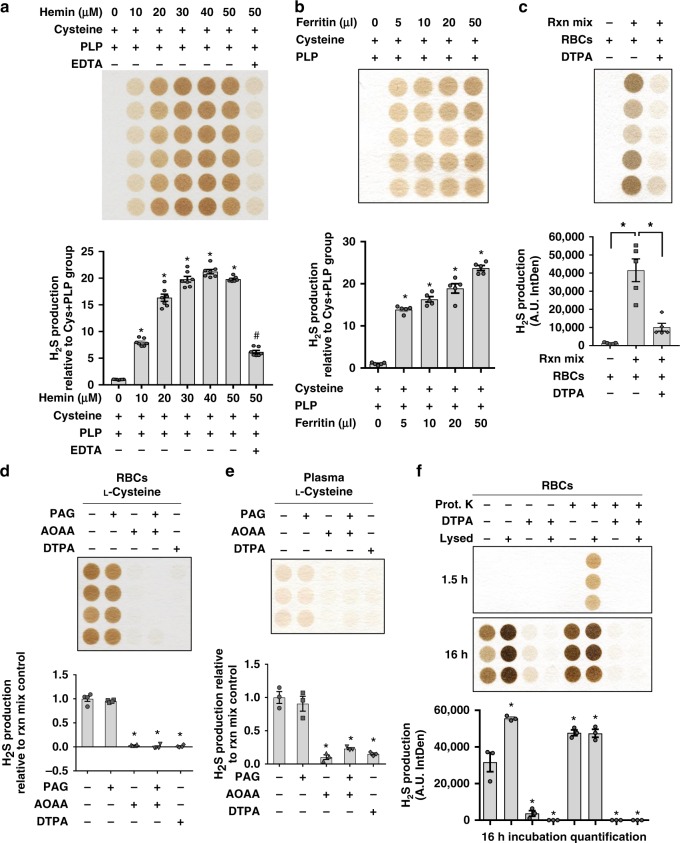
Heme-bound iron catalyzes H_2_S production in vitro and in blood/plasma ex vivo. **a**, **b** Hemin (**a**; *n* = 6/group) and ferritin (**b**; *n* = 5/group) dose dependently catalyze H_2_S production from l-cysteine and PLP. Asterisk indicates the significance of the difference versus the l-cysteine and PLP control group; *P < 0.05. **c** Iron-dependent H_2_S production from RBC lysate ± l-cysteine and PLP reaction mix, ±DTPA; *n* = 5/group. Asterisk indicates the significance of the difference between indicated groups; *P < 0.05. **d**, **e** H_2_S production from RBC lysate (**d**; *n* = 4/group) or plasma (**e**; *n* = 3/group) ± theoretical inhibitors PAG and AOAA and ±iron chelator DTPA with the addition of l-cysteine and PLP. Asterisk indicates the significance of the difference versus the l-cysteine and PLP control group; *P *<* 0.05. **f** H_2_S production from the reaction mixture of whole or lysed RBCs, l-cysteine, and PLP ± pretreatment of DTPA and ±Prot. K); *n* = 3/group. Asterisk indicates the significance of the difference versus non-lysed RBCs with no DTPA or Prot. K pretreatment; *P < 0.05. All data were presented as mean ± SEM

The majority of iron in the body is bound to heme, heme-like structures, and/or proteins^38^. It is undetermined whether these biologically relevant forms of iron catalyze H_2_S production similar to free iron. Hemin (Supplementary Fig. 6A), a ferric protoporphyrin-IX group formed during RBC and heme turnover^39^, and ferritin, the major iron-storage protein complex in tissues and circulation^40^, dose dependently increased H_2_S production from L-Cys in the presence of PLP (Fig. 6a, b), which was dampened by EDTA (Fig. 6a). SIFT-MS headspace analysis confirmed hemin and ferritin catalyzed H_2_S production (Supplementary Fig. 6B). Similar to free Fe^3+^-driven H_2_S production (Fig. 2d), normoxic 20% O_2_ optimized hemin- and ferritin-catalyzed H_2_S production compared with hypoxic conditions (Supplementary Fig. 6C). H_2_S production in the circulation, specifically RBCs and plasma, was dependent on PLP (Supplementary Fig. 6D) and L-Cys, but not l-homocysteine (Supplementary Fig. 6E) and iron (Fig. 6c). Iron chelation via EDTA or diethylenetriaminepentaacetic acid (DTPA) inhibited H_2_S production in the RBCs and plasma (Fig. 6c, d, e, f), but not in the liver (Supplementary Fig. 6F). Iron is thus dispensable for enzymatic production in the liver, which is heavily CGL-driven (Fig. 1c), but not for non-enzymatic production in the circulation.

To further confirm iron-catalyzed H_2_S production in the blood and plasma as non-enzymatic, we utilized several pharmacologic, biochemical, and genetic tools to rule out CBS, CGL, and/or 3-MST activity. Small-molecule inhibitors of CGL and CBS were first used to examine inhibition of potential enzymatic H_2_S production in RBCs (Fig. 6d and Supplementary Fig. 6G) and plasma (Fig. 6e). PAG, which inhibited enzymatic hepatic H_2_S production (Supplementary Fig. 5B) but not non-enzymatic H_2_S production in vitro (Fig. 5b), had no effect on RBC (Fig. 6d) or plasma (Fig. 6e) H_2_S production. Similarly, blood and plasma from *CGL* KO mice had no deficiencies in H_2_S production compared with WT mice (Fig. 1d and Supplementary Fig. 6E). These findings are consistent with the absence of CGL, and thus CGL-derived H_2_S, in RBCs^41^. To rule out CBS, we first used AOAA, a purported general inhibitor of PLP-dependent enzymes, including CGL, CBS, and CAT^33^. AOAA abolished H_2_S production in RBCs (Fig. 6d and Supplementary Fig. 6G) and plasma (Fig. 6e). However, as AOAA inhibits non-enzymatic H_2_S production in vitro (Fig. 5b), it cannot be used to accurately rule out CBS or enzymatic-mediated H_2_S production in the blood and serum. With this, we next utilized d-cysteine, which acts equally to L-Cys as a substrate for non-enzymatic H_2_S production (Fig. 4b) but cannot be used for enzymatic production (Supplementary Fig. 4A). d-Cysteine readily formed H_2_S in RBCs and this was abolished with the addition of AOAA and DPTA (Supplementary Fig. 6G). Thus, the iron dependence for H_2_S production in the blood is not related to the regulatory heme-containing prosthetic group on CBS^42^ but via the non-enzymatic iron/PLP-catalyzed chemical reaction. To further rule out CAT/3-MST or other possible H_2_S-generating enzymes, proteinase K treatment of RBCs increased H_2_S production, which was abolished by DTPA (Fig. 6f). Taken together, H_2_S production in the blood and plasma is predominantly non-enzymatic and requires iron- and VitB_6_-coordinated catalysis of cysteine.

H_2_S exhibits toxic and beneficial characteristics, with the regulation and health-related impacts of its endogenous enzymatic production well understood^6,7,11^. However, with non-enzymatic iron-catalyzed H_2_S production, questions remain regarding regulation and biological consequences. Iron-catalyzed production of reactive oxygen species (ROS) drives the pathophysiology of iron-overload disorders and hemolytic anemias, and is exacerbated when free iron is released^43^. We hypothesized that under similar states of hemolytic anemia or dysregulated iron homeostasis, H_2_S production is altered and this contributes to the pathophysiology associated with these disorders. To begin testing this hypothesis, we examined the impact of whole vs. lysed RBCs on H_2_S production. Lysed RBCs increased iron-dependent H_2_S production capacity compared with non-lysed RBCs (Fig. 6f). Similarly, degradation of RBC proteins via Prot. K treatment increased iron-dependent H_2_S production in both whole and lysed RBCs (Fig. 6f). H_2_S production was greatest in lysed RBCs treated with Prot. K and this was dependent on iron (Fig. 6f). Catalytic activity of free Fe^3+^ was greater than the bound hemin form when compared in equimolar chemical reactions (Supplementary Fig. 6H). Thus, the integrity of RBCs and protein homeostasis regulate iron-catalyzed H_2_S, most likely through sequestration of iron into the bound vs. free faction, with the latter having increased catalytic potential. We additionally tested the effect of protein homeostasis on non-enzymatic H_2_S production in the brain (Supplementary Fig. 6I). H_2_S production was increased in whole brain extracts treated with Prot. K and was dependent on iron (Supplementary Fig. 6I). Thus, the regulation of protein homeostasis and cellular integrity on the iron-driven non-enzymatic H_2_S production is potentially not restricted to the circulation but common throughout tissues.

## Discussion

**Fig. 7:**
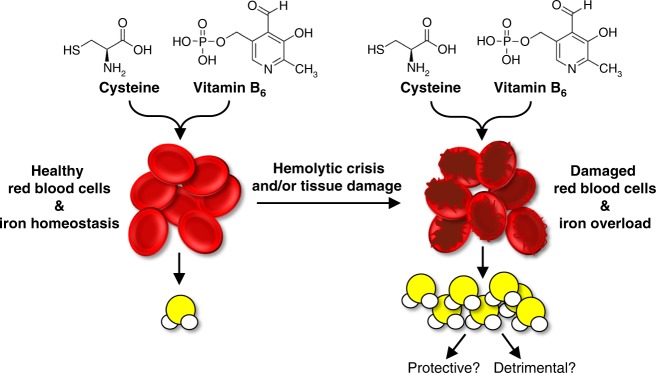
Experimental model of red blood cell state and tissue integrity impacting iron-catalyzed non-enzymatic H_2_S production. Iron in red blood cells and tissues catalyzes the production of H_2_S in coordination with VitB_6_ from the sulfur-containing amino acid cysteine at physiological temperatures, pH, and oxygen conditions. Multiple biologically relevant forms of VitB_6,_ PLP or pyridoxine, and iron, free or bound Fe^3+^/Fe^2+^, served as catalysts. Upon hemolysis, tissue damage, and/or degradation of iron- and heme-containing proteins, the catalytic potential of iron is increased and more H_2_S is produced. The biological significances of this increased H_2_S production, particularly in the context of hemolytic anemias and crises, are yet to be determined

In the current study, we examined enzymatic and non-enzymatic H_2_S production in organs and circulation. We determined enzymatic production is primarily driven by CGL and predominates in the liver and kidney, whereas non-enzymatic production prevails in the majority of other tissues. Non-enzymatic production of H_2_S was partial for cysteine as substrate and catalyzed by coordinated activities of VitB_6_ and iron at physiological temperatures, pH, and oxygen. Multiple biologically relevant forms of VitB_6_ and iron served as catalysts, whereas the state of RBCs and protein homeostasis impacted H_2_S production capacity. These results are summarized in Fig. 7.

Biological contexts and implications for the non-enzymatic production of H_2_S are not known, particularly if this reaction serves beneficial or detrimental roles. Nevertheless, the dietary, genetic, and hemolytic factors affecting VitB_6_ and iron bioavailability likely impact non-enzymatically produced H_2_S and this production may influence the physiological outcomes related to these factors. In the following discussion, we place our findings into biological contexts and established enzymatic and non-enzymatic sulfide-related fields.

Abnormal iron accumulation is associated with Alzheimer’s^44^, Parkinson’s^45^, hemolytic^46^, and hemochromatosis diseases^47^. Iron catalyzes the formation of ROS via the Fenton reaction^48^. Although iron-catalyzed ROS serves beneficial functions such as signal transduction^49^, it also damages macromolecules^50^. Similar to ROS, H_2_S plays beneficial and detrimental roles dependent on dosage, tissue, and exposure route^51^. Controlled enzymatic H_2_S production is generally beneficial^52^. However, the consequences of non-enzymatic and theoretically less regulated H_2_S production are not known. Interestingly, H_2_S prevents the formation of ROS-induced protein aggregation found in neurodegenerative tissues^53^ and prevents neurocognitive decline^54^. In addition, deficient VitB_6_ status, of which we show suppresses iron-catalyzed H_2_S production, is a biomarker for neurocognitive decline in humans^55^. Thus, iron may catalyze the production of harmful ROS concurrently with protective H_2_S and VitB_6_ status may shift the balance away from ROS to H_2_S. This may explain why chelation therapies targeting iron in neurodegenerative diseases have delivered mixed results^56^. Future work entails examining downstream effects of this non-enzymatic production, such as posttranslational thiol-sulfhydration, tissue histology, and interventions to control the reaction under pathological settings of iron dysregulation.

Aerobic exercise provides metabolic and physiological benefits, but also induces RBC hemolysis^57^. This is due to increased oxygen demands, osmotic perturbations, and compression of capillaries in muscles damaging RBCs^57^. When comparing non-impact aerobic exercises vs. high-impact running, it was discovered that running produced more hemolysis and plasma-free hemoglobin due to foot strike and circulatory trauma^57^. As H_2_S provides similar benefits to exercise, such as angiogenesis^16^, one must ponder the extent non-enzymatic iron-catalyzed H_2_S provides in generating the benefits of exercise and running.

Enzymatic H_2_S production in solid tissues has been known for over 20 years^58–60^ and its VitB_6_-dependent mechanisms for over a decade^61,62^. However, H_2_S levels and metabolism in blood and circulation are currently not well understood^41,63^. Although circulating levels of free or bound H_2_S can be used as cardiovascular risk factors within individual trials^64^, the source for this H_2_S is unclear. Previous studies suggest CBS and CGL are secreted into the bloodstream by the liver and vascular endothelial cells^65^, whereas others propose production in the blood is derived via 3-MST^41^. However, in our current study, we show H_2_S production derived from cysteine in the plasma/serum and RBCs is non-enzymatic and involves iron and VitB_6_. Although our study does not rule out the contributions of CBS, CGL, and 3-MST, it sets forth the theory that basal amounts of H_2_S in the bloodstream are of non-enzymatic origin. Interestingly, this is not the first study to establish iron’s role in blood H_2_S metabolism. Although we report iron catalyzing the production of H_2_S, others show iron oxidizes and removes H_2_S from blood^41,66^. Thus, iron has several roles in H_2_S biogenesis, metabolism, and detoxification.

We were initially puzzled that other SAAs, particularly homocysteine, did not serve as readily available H_2_S-producing substrates compared with cysteine for the chemical reaction. Homocysteine has only one extra carbon in the γ-position compared with cysteine and serves as substrate for enzymatic H_2_S production^6^. With no stringent steric requirements in the chemical reaction compared with the enzymatic reaction, it is unclear why homocysteine did not produce H_2_S as rapidly as cysteine. An explanation is that PLP is expected to react with homocysteine and cysteine to form six-member tetrahydrothiazine and five-member thiazolidine rings^37^, respectively. Consequently, the tetrahydrothiazine six-member ring is more thermodynamically stable compared with the five-member thiazolidine ring. Thus, it requires more time/energy for iron to break the six-member ring and/or access the exposed thiol from the broken bond between the sulfhydryl group and the imine carbon from homocysteine compared with cysteine.

There exists the possibility of outcompeting and side reactions resulting in additional products and impacting the H_2_S yield. Such products, although not detected in the methods utilized here, could be non-volatile sulfur-containing species that remain in solution, such as cysteine hydropersulfide and polysulfides. Due to the many oxidation states of sulfur^67^, Fe^3+^-mediated oxidation could result in numerous additional sulfur species. Furthermore, H_2_S produced from the reaction may react with iron to form acid-labile iron-sulfide precipitates^68^ and lower the detected H_2_S yield. Future studies are needed to capture all products pertaining to cysteine, VitB_6_, and iron, and how these impact basic biological process and pathophysiological outcomes.

Our results also provide insight regarding blood and tissue collection for downstream H_2_S production analysis. Blood collection tubes contain anti-coagulants, including EDTA. Our results indicate this metal chelator suppresses iron-catalyzed H_2_S production. Thus, EDTA use during sample collection can result in underestimating total H_2_S production capacity. Conversely, EDTA use would facilitate a focused analysis of enzymatic H_2_S production. In addition, we found the reductant DTT serves as substrate for H_2_S production via Fe^3+^ catalysis. This data could be used by investigators to avoid false positives when examining total sulfide pools in biological systems, particularly those that contain iron, when initial steps require the release of bound sulfane sulfur via the addition of a reductant^69^. Thus, non-sulfur-containing reductants, such as tris(2-carboxyethyl)phosphine (TCEP), may lessen the occurrence of false-positive H_2_S detection.

In summary, we reveal a novel chemistry occurring under physiological conditions for iron and VitB_6_ coordinately and non-enzymatically catalyzing the production of H_2_S from cysteine. We show the importance of free vs. bound states of iron and the integrity of RBCs in controlling the robustness of this reaction. Ultimately, this study establishes a new area in hematological research with future directions to decipher downstream effects of altered iron-catalyzed H_2_S in tissues most affected by hemolytic crisis, hemorrhage, and iron overload, and to develop cysteine- and VitB_6_-based interventions for the treatment of these disorders.

## Methods

### Animal studies

All experiments were performed with the approval of the Institutional Animal Care and Use Committee from the Cleveland Clinic Lerner Research Institute, protocol number 2016–1778. Animals were bred and maintained under standard housing conditions in the Cleveland Clinic Biological Resource Unit with ad libitum access to food (Envigo #2918) and water, 12 h light/12 h dark cycles, temperature between 20 and 23 °C with 30–70% relative humidity, and weaned between 3 and 4 weeks of age. Tissues from mice used in Fig. 1c, d and Supplementary Fig. 1A-C were 6-week-old and that used in Fig. 6f and Supplementary Fig. 6D were 1-year-old *CGL* WT and KO mice on a mixed 129/C57BL/6 background as previously described^15,70^, and those in Fig. 6c were 6-month-old C57BL/6 male mice. Tissues used in the remaining figures are adult 6–8-month-old C57BL/6J male mice (Jackson Laboratory).

### RBC hemolysis

Mice were anesthetized with isoflurane and blood collected via retro-orbital bleed and immediately placed into lithium-heparin-coated capillary tubes (Terumo #T-MLH). Tubes were centrifuged to separate RBCs from the plasma. After removing the plasma supernatant, equal volume of PBS was added to the packed RBC pellet and mixed gently to resuspend the RBCs. The RBC suspension was then split in half, with one half flash frozen in liquid nitrogen and then thawed at room temperature for a total of three rounds to facilitate hemolysis. The samples were then spun down and the RBC lysates (supernatant) were ready for further determination of H_2_S production.

### Lead acetate/lead sulfide method for H_2_S determination

H_2_S production was measured using the lead acetate/lead sulfide method^21^. Briefly, 150 µL of reaction mixture was performed in either PBS or cell culture media DMEM or DMEM/F12. The reaction mixture contained 10 mM L-Cys (Sigma #168149) or other SAAs d-cysteine (Sigma #30095), l-homocysteine (Sigma #69453), dl-homocysteine (Sigma #H4628), l-methionine (Sigma #64319), glutathione (Sigma #G6013), and NAC (Sigma #A7250) as substrate, and 1 mM PLP (Sigma #9255) or pyridoxal (Sigma #271748) as cofactor, and placed in 96-well assay plates and incubated with the addition of FeCl_3_ (Alfa Aesar #12357), FeCl_2_ (Alfa Aesar #31141), hemin (Sigma #51290), ferritin (Sigma #F4503), or other metal irons Mn^2+^ (Sigma #203724), Zn^2+^ (LabChem #LC270701), Cu^2+^ (Sigma #209198), Pb^2+^ (Sigma #316512), Al^3+^ (Sigma #294713), Ca^2+^ (Sigma #223506), and Mg^2+^ (Sigma #M-9397) as non-tissue-derived catalysts. The concentration of the catalysts used was 50 µM or as indicated in the figures. Other organic sulfur compounds, including artificial garlic oil blend (Sigma W530316) and two major garlic components, DATS (Sigma SMB00289) and DADS (Sigma SMB00378), DTT (Sigma 43815) and 2ME (Fisher Scientific C3446I), were also tested as substrates. The garlic oil blend was added into the chemical reaction at 1000-fold dilution and votexed before the incubation, and others were used at 10 mM working concentrations. Filter paper, embedded with lead acetate (Sigma #316512), was placed above the assay plate and incubated at 37 °C air chamber between 1 and 24 h until a desirable amount of lead sulfide was detected for proper quantification. To determine the H_2_S production under anaerobic condition, the chemical mixture of L-cys/PLP/Fe^3+^ in PBS was run in 96-well format in a hypoxia/anoxia chamber (COY Laboratory Products, Inc.) with the oxygen (O_2_) level set to 0% with nitrogen (N_2_) resulting in an actual hypoxic environment of 0.1–0.2% O_2_. Similar procedures were used when measuring the H_2_S production capacity of tissues, blood, and plasma. In these cases, 100 µg tissue homogenate in passive lysis buffer (Promega #1941) or 10–20 µL of whole RBCs or RBC lysate, or plasma/serum was added to the 150 µL reaction mixture containing 10 mM L-Cys and 1 mM PLP, followed by the same protocol as described above for determination of H_2_S production. Tissue extracts used in Fig. 1e were pretreated ex vivo with Proteinase K (Prot. K) (Sigma # 3115879001) in 37 °C water bath for 1 h, to remove all potential enzymatic activity prior to performing H_2_S production. In reactions pretreated with the inhibitors, 10 mM EDTA (Sigma #5134), 100 µM DTPA (Sigma #D6518), 2 mM PAG (Sigma #P7888), or 2 mM AOAA (Sigma #C13408) were added to the reaction mixture prior to the addition of the chemical or tissue catalyst.

### Head space measurement of H_2_S production

H_2_S production from reaction mixture of L-Cys, PLP, and iron was quantified using two instruments: (1) the Jerome J605 as shown in Supplementary Fig. 3B, a gold film H_2_S analyzer^44^, and (2) the SIFT-MS (VOICE200R SIFT-MS instrument, Syft Technologies, Ltd, Christchurch, New Zealand), measuring the product ions generated in the reaction of H_3_O^+^ in H_3_O^+^ + H_2_S ↔ H_3_S^+^ + H_2_O, being the mass-to-charge (*m*/*z*) 35 product ion^26,27^. Briefly, 1 mL of reaction mixture containing supraphysiological concentrations of L-Cys (10 mM), PLP (1 mM), and Fe^3+^ (50 µM), or physiological concentrations of L-Cys (500 µM), PLP (500 nM), and Fe^3+^ (50 µM) were placed in headspace vials (for H_2_S detection in the Jerome J605, Wheaton #225277 6 mL headspace vials; for H_2_S detection in the SYFT-MS, Agilent 20 mL headspace vials) and sealed with aluminum seal caps containing a polytetrafluoroethylene (PTFE)/silicone septa (Restek #21763). Reaction mixtures were incubated at 37 °C for a desired amount of time (for H_2_S detection in the Jerome J605, 1 h with supraphysiological reaction mixture and 6 h with physiological reaction mixture; for H_2_S detection in the SIFT-MS, 30 min at 37 °C). Afterwards, headspace air was sampled via needle and syringe, 1 mL for Jermone J605 analysis and 15 mL for SIFT-MS, and injected directly into the instrument for detection. The concentration of H_2_S with displayed at p.p.b. for the Jerome J605 and at p.p.m. for SIFT-MS. Headspace air was also sampled and injected to SIFT-MS, measuring the full mass spectrum over the range of *m*/*z* shown in H_3_O^+^ reagent ion.

### H_2_S and pyruvate production with fluorescent probe detection

Kinetic H_2_S production was measured using AzMC (Sigma #802409), which is a fluorogenic probe selectively reduced in the presence of H_2_S producing 7-amino-4-methylcoumarin^29^ and detectable with excitation wavelength Exc 350 nm and emission wavelength Emi 445 nm. Briefly, 150 µL of reaction mixtures containing combinations of 10 mM L-Cys, 1 mM PLP, and 50 µM Fe^3+^ in the presence of 100 μM AzMC probe were placed in 96-well plates and analyzed at various timepoints on SpectraMax i3 multimode plate reader through bottom reading (37 °C, *λ*_Exc/Emi_ = 350/445 nm, photomultiplier tube (PMT): medium) with 10 min interval. Similarly, kinetic pyruvate production was measured using a plate-based pyruvate assay kit (Abcam #ab65342). Briefly, in a clear-bottom 96-well plate, 50 µL of H_2_S reaction mixture (10 mM L-cys, 1 mM PLP ± 50 µM FeCl_3_ in PBS) was mixed with 50 µL of pyruvate colorimetric reaction mixture. The pyruvate product was analyzed on SpectraMax i3 multimode plate reader through bottom reading (37 °C, *λ* = 570 nm) with 30 min time interval.

### LC-MS/MS analysis of standards, intermediates, and products

Chemical reactions containing individual L-Cys (10 mM), PLP (1 mM), or FeCl_3_ (50 µM), or a mixture of the above compounds were incubated at 37 °C for 3 h prior to freezing at −20 °C overnight. A full reaction mixture without 37 °C incubation was put in freezer at −20 °C immediately, serving as a negative control to the full reaction mixture with 37 °C incubation. All samples were submitted for untargeted LC-MS/MS analysis. The samples were thawed, placed in high-performance LC (HPLC) vials, and the vials were place in the chilled autosampler (4 °C). The LC-MS instrument was configured with an HPLC containing two Shimadzu LC-20AD Pumps, DGU-203R Autosampler, and CBM-20A Communications Module, and a time-of-flight (TOF) high-resolution accurate-mass spectrometer (SCIEX TripleTOF 5600). The HPLC column was a Phenomenex Prodigy 5μ ODS, 2.0 × 150 mm reversed-phase column. Sample injection volume was 5 μL and the eluent flow rate was 0.2 mL/min. The HPLC used a gradient formulated from different proportions of Eluent A (water + 0.2% formic acid) and Eluent B (methanol + 0.2% formic acid). The gradient began at 100% Eluent A and remained there for 3 min. Next, a linear gradient from 100% Eluent A to 100% Eluent B over 6 min was formulated and the system then remained at 100% Eluent B for 6 min. The system was then returned to 100% Eluent A for 10 min, re-equilibrating the chromatographic column in preparation for the next injection. Each sample was injected four times to undergo four different MS analyses as follows: (1) a full-scan positive ion MS TOF (20–750 *m*/*z*) survey scan was collected and used to trigger data-dependent MS/MS analysis of the most intense ion from the survey scan. (2) This TOF-IDA (Information-Dependent Acquisition) analysis was repeated in the negative ion mode. (3) Full-scan positive ion MS TOF (40–750 *m*/*z*) survey scans were followed by MS/MSall (SWATH) data collection, using 18 product ion scans with 40 *m*/*z* widths to cover the entire survey scan mass range. (4) This TOF-SWATH (Sequential Window Acquisition of all Theoretical fragment ion spectra) analysis was repeated in the negative ion mode. Data were analyzed by generating full scan extracted ion chromatograms (XICs) generated from the formula weights of the compounds expected to be present using positive/negative ion TOF analysis. The XICs for L-Cys and cysteine-aldimine/quinonoid/thiazolidine were analyzed at positive ion TOF with mass of 122.0166 ± 0.0029 Da and 351.010 ± 0.006 Da, respectively. The XICs for aldimine and pyruvate were analyzed at negative ion TOF with mass from 314.75 to 315.25 Da and from 86.75 to 87.25 Da, respectively.

### UV/Vis measurement of PLP absorbance

One hundred and fifty microliters of PBS containing 1 mM PLP in the absence or presence of the SAAs previously listed was placed in 96-well assay plates and analyzed on a SpectraMax i3 multimode plate reader at an absorbance spectrum between 280 and 460 nm at room temperature.

### Immunoblot and Coomassie staining for protein expression

Protein analysis was performed via western blotting on tissue homogenates in passive lysis buffer (Promega), separated by SDS-polyacrylamide gel electrophoresis, transferred to polyvinylidene difluoride membrane (Whatman) and blotted for CGL (ab151769 Abcam), CBS (ab135626 Abcam), MST (#85211 Abcam), or α-Tubulin (#4074 Abcam), followed by horseradish peroxidase-conjugated secondary anti-rabbit antibody (#97051 Abcam). Proteins were visualized using SuperSignal West Femto Maximum Sensitivty Substrate (Thermo Scientific #34096) on an Amersham Imager 600 (General Electric) and sizes determined using the PageRuler Plus Prestained (26619 Thermo Fisher). After blotting and washing, the membranes were stained using Coomassie blue, to determine the relatively equal amount of tissue proteins loaded.

### Statistics and reproducibility

Data are displayed as means ± SD or means ± SEM with *n*-values between 3 and 6 as indicated in the figure legends. The majority of the experiments were performed at least twice independently with multiple technical replicates performed for each independent experiment. Statistical significance was assessed in GraphPad Prism and/or Microsoft Excel using Student’s *t*-tests to compare values between two specific groups and one-way analysis of variance with a Tukey’s multiple comparison test for comparing more than two groups in a single data set. A *P*-value of 0.05 or less was deemed statistically significant and statistical details are found in the figures and figure legends. Quantifications of lead acetate/lead sulfide H_2_S production capacity images were performed using the IntDen measurement function in ImageJ software and normalized to the respective control group after subtracting the background.

### Reporting summary

Further information on research design is available in the Nature Research Reporting Summary linked to this article.

## Supplementary information

Supplemental Figures and Table

Reporting Summary

## Data Availability

The authors declare that the majority of the data supporting the findings of this study are available within the paper and its supplementary information files. Additionally, raw data are available from the corresponding author upon reasonable request.
