# Supplementary material for: Non-enzymatic hydrogen sulfide production from cysteine in blood is catalyzed by iron and vitamin B6
Source: Commun Biol. 2019 May 21;2:194. doi: 10.1038/s42003-019-0431-5 (PMC6529520; doi:10.1038/s42003-019-0431-5)
Supplement: Supplementary file 1 — Supplemental Figures and Table [file 42003_2019_431_MOESM1_ESM.pdf]

Supplementary Figures

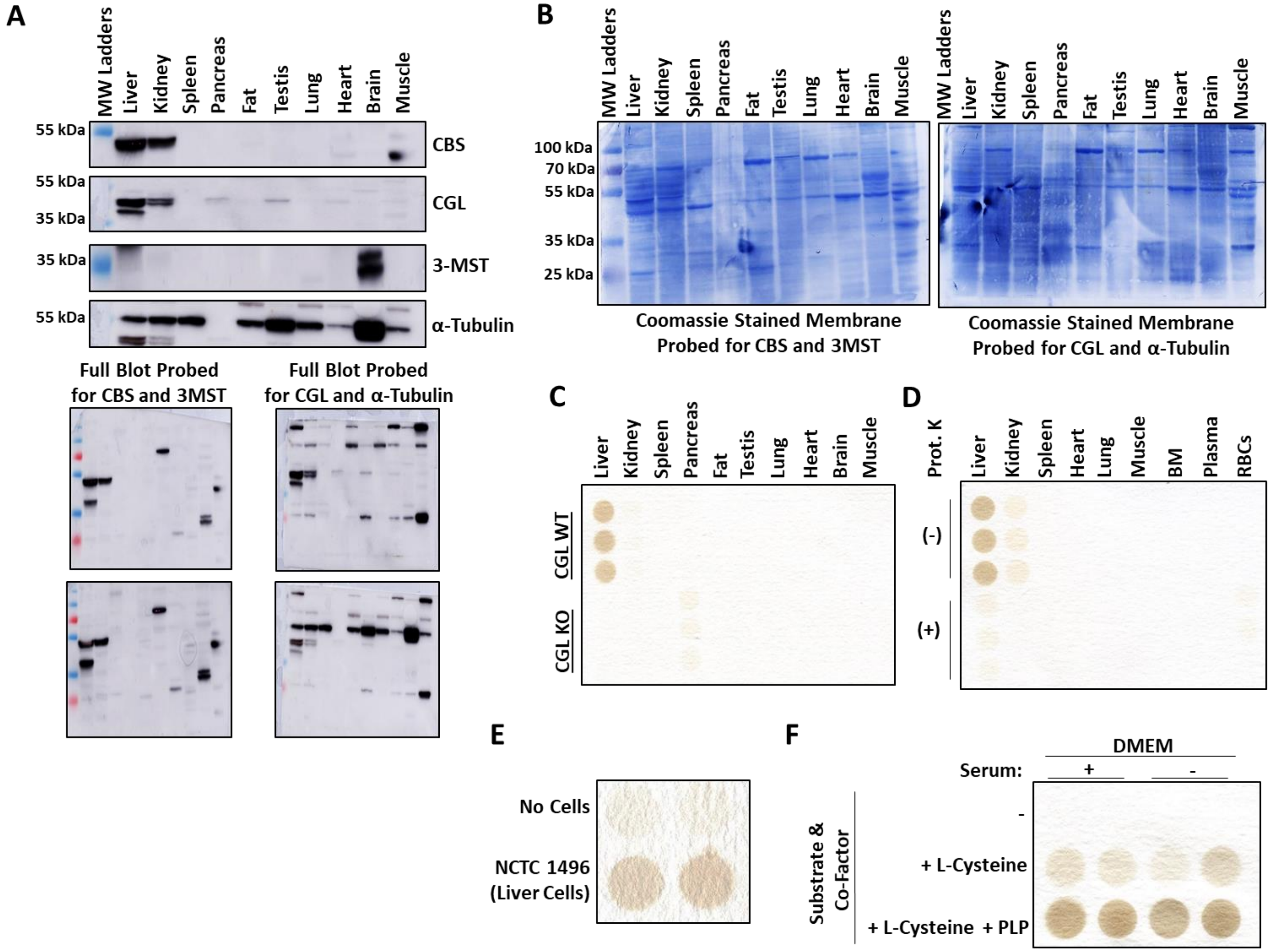

Supplementary Figure 1: Tissue specific enzymatic and non-enzymatic H_2S production. (**A and B**): Western immunoblots of CBS, CGL, 3-MST, and α-Tubulin in tissue extracts from *CGL* WT mice. The full uncropped blot images probed for CBS, CGL, 3-MST, and α-Tubulin are placed at the bottom (**A**). The membranes probed with above protein antibodies were further stained with coomassie blue to indicate relative protein loading (**B**). (**C**) Similar to Figure 1C, H_2S production from tissue extracts from *CGL* WT and *CGL* KO mice in the presence of L-cysteine and PLP; n = 3/group. Exposure time is shorter (1-hr) than in Figure 1C to prevent saturation. (**D**) Similar to Figure 1E, H_2S production capacity of tissue extracts, plasma, and RBCs from *CGL* WT mice in the presence of L-cysteine and PLP ± Prot. K pretreatment; n = 3/group. Exposure time is shorter (1.5-hr) than in Figure 1E to prevent saturation. (**E**) H_2S production from control cell-free wells or wells with live NCTC 1496 mouse liver cells with the addition of L-cysteine and PLP in the cell culture media; n = 2/group. Note production is detected even in the cell-free control wells. (**F**) H_2S production mediated in cell-free DMEM media in the presence ± serum, ± L-cysteine and ± PLP; n = 2/group.

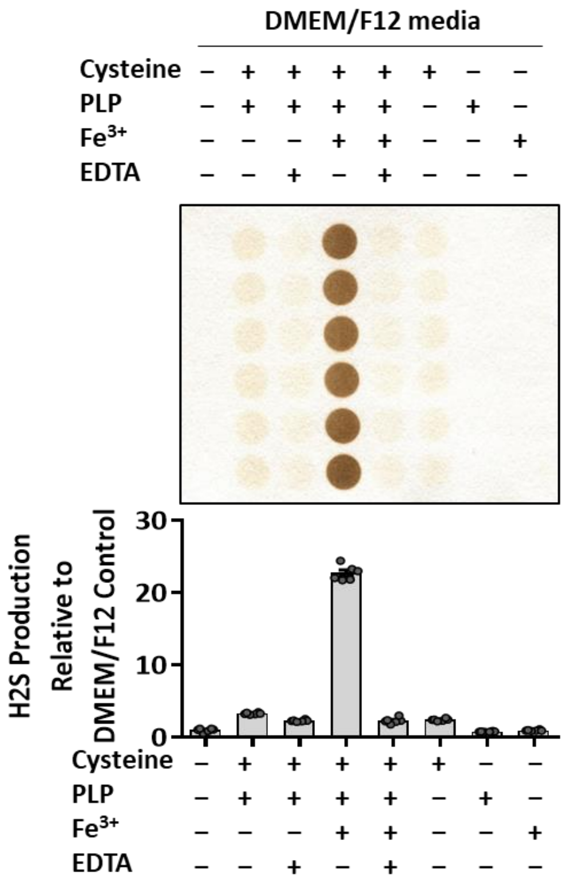

Supplementary Figure 2: Metal ions induce non-enzymatic H_2S production. H_2S production mediated in DMEM/F12 media in the presence ± L-cysteine, ± PLP, and ± Fe^{3+} with ± EDTA pretreatment; n = 6/group. All data were presented as mean ± SEM.

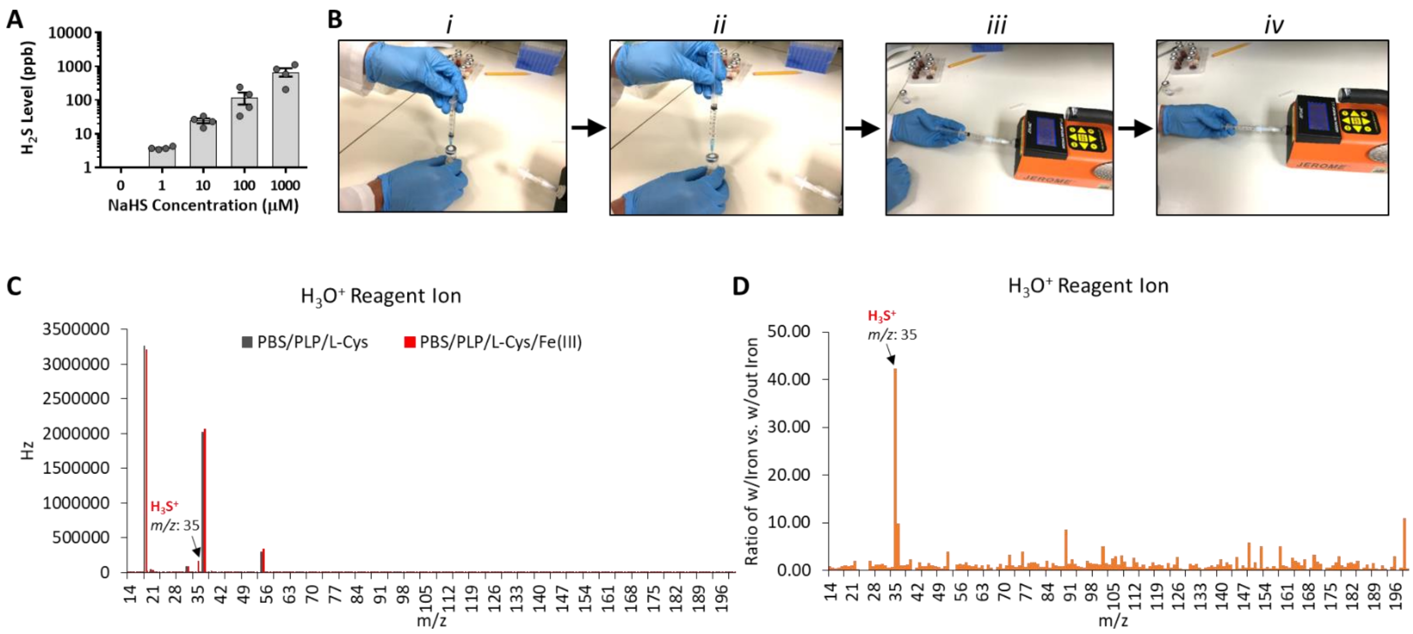

Supplementary Figure 3: H_2S detection by the Jerome J605 gold film H_2S analyzer and SYFT-MS instrument. (**A**) H_2S headspace levels (parts per billion; ppb) after 1 hr incubation at 37°C of various concentrations of NaHS in 1 mL pf PBS in a 6 mL Wheaton headspace vial; n = 2/group. Data were presented as mean ± SEM. (**B**) Pictorial description for the use of the Jerome J605 gold film H_2S analyzer for measuring H_2S headspace concentrations from 6 mL Wheaton headspace vials. 1 mL of headspace air is removed with a syringe and needle from the vials after incubation of the reactions mixtures for a given period of time and then directly inserting into custom made Tygon tube adapter attached to the Jerome J605 and compressing the syringe plunger to dispel the air into the analyzer which then gives an immediate H_2S reading. **(C)** Full mass spectra over the range of mass-to-charge (*m/z*) shown in the H_3O^+ reagent ion in the selected ion flow tube mass spectrometry (SYFT-MS) instrument, showing the precursors and products from the two reaction mixtures (w/ Fe^{3+} vs. w/out Fe^{3+}). (**D**)The product ratio of the full mass spectra from the two reaction mixtures (w/ Fe^{3+} vs. w/out Fe^{3+}). Note the major peak at *m/z* 35 for H_3S^+ representing H_2S after it reacts with the H_3O^+ SIFT-MS reagent ion is the predominate difference between the two reaction mixtures.

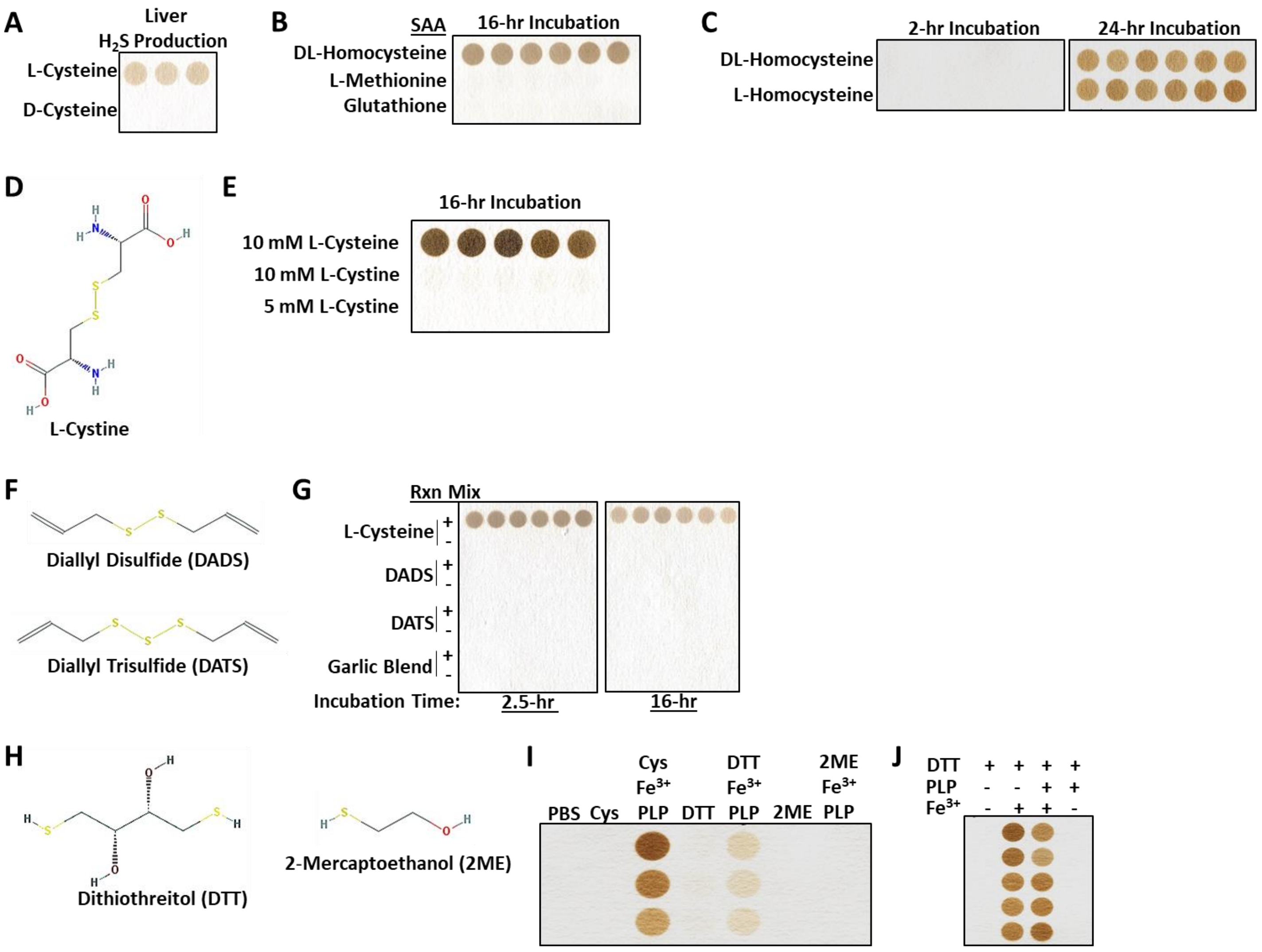

Supplementary Figure 4: Selectivity of substrates and co-factors for enzymatic and non-enzymatic H_2S production. (**A**) Selectivity of L-cysteine versus D-cysteine as substrate for enzymatic H_2S production catalyzed in mouse liver extract; n = 3/group. (**B**) H_2S production capacity after 16-hr incubation with DL-homocysteine, L-methionine, and glutathione as substrates catalyzed by Fe^{3+} and PLP; n = 6/group. (**C**) Selectivity of DL-homocysteine versus L-homocysteine as substrate for non-enzymatic H_2S production after 2- and 16-hr incubation exposures at 37°C; n = 6/group. (**D**) Chemical structure of L-Cystine. (**E**) H_2S production from L-Cystine in reaction mixture of PLP and Fe^{3+}; n = 5/group. (**F**) Chemical structure of major garlic components, Diallyl Disulfide (DADS) and Diallyl Trisulfide (DATS). (**G**) H_2S production from garlic blend, major garlic components (DADS and DATS) in reaction mixture of PLP and Fe^{3+} with 2.5- and 16-hr incubation exposures at 37°C; n = 6/group. (**H**) Chemical structure of Dithiothreitol (DTT) and 2-Mercaptoethanol (2ME). (**I**) H_2S production from DTT and 2ME in reaction mixture of PLP and Fe^{3+}; n = 3/group. (**J**) H_2S production from DTT in PBS in the presence ± PLP and ± Fe^{3+}; n = 6/group.

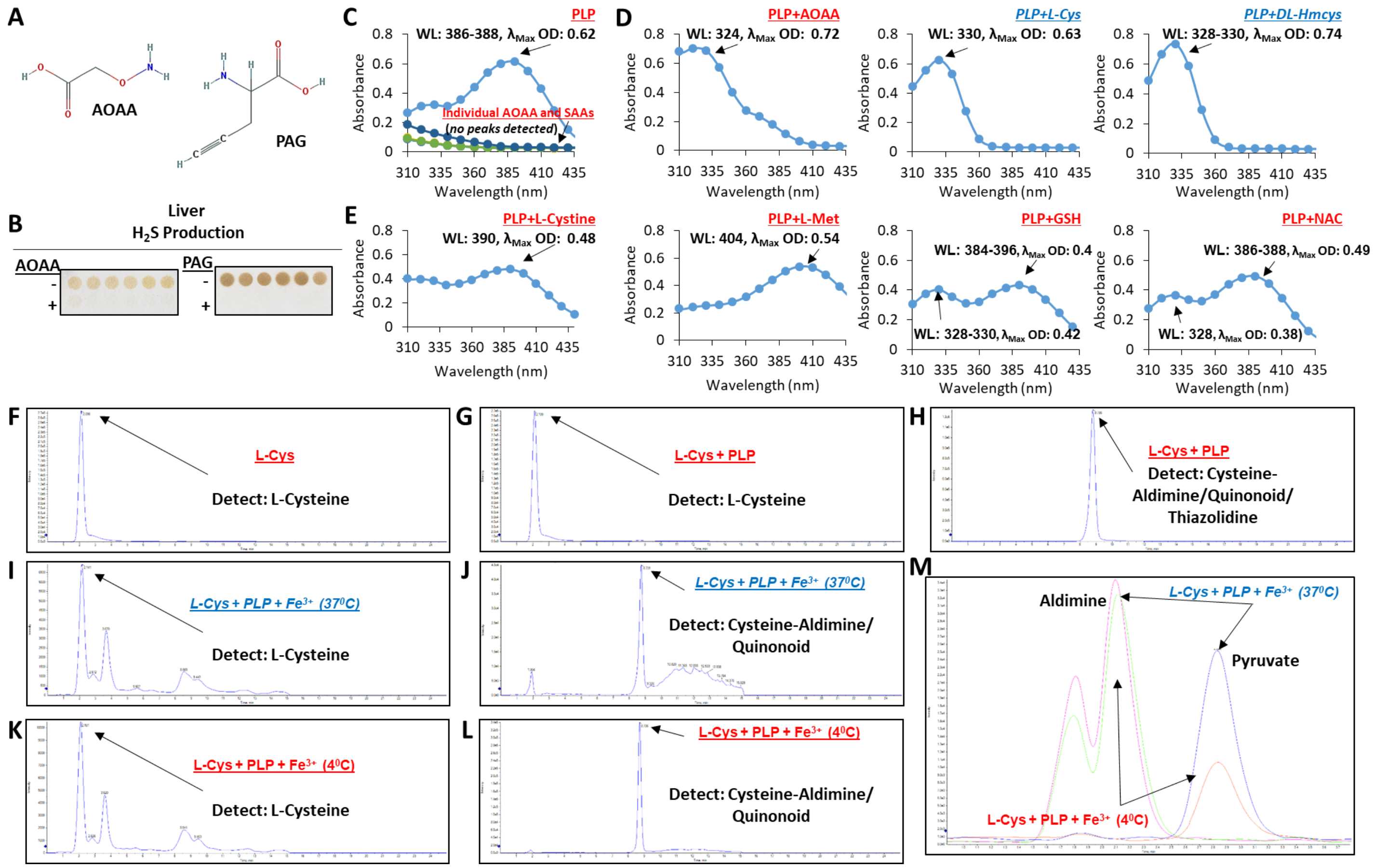

Supplementary Figure 5: Spectroscopic analysis of PLP and SAA interactions. (**A**) Chemical structures of PAG and AOAA. (**B**) The inhibition of enzymatic H_2S production in mouse liver extract by PAG and AOAA; n = 6/group. (**C**) Absorbance of PLP and individual tested sulfur containing amino acids (SAAs) in the spectrum of 310 nm to 435 nm in PBS at 37°C. (**D and E**) Absorbance of the PLP and SAAs/AOAA mixtures in the spectrum of 310 nm to 435 nm. The SAAs selected as substrate for non-enzymatic H_2S production are highlighted in blue italic, and the SAAs highlighted in red indicate their unavailability as substrate. (**D**) AOAA, cysteine, and homocysteine form a single peak absorbance at ~330 nm when incubated with PLP, indicative of the Schiff base. (**E**) Cystine, Methionine, glutathione, and NAC do not form a single peak Schiff base indicating single peak absorbance at ~330 nm when incubated with PLP. (**F-M**) Mass spectrometry full scan extracted ion chromatograms (XICs) generated from the formula weights of the compounds expected to be present using positive/negative ion time-of-flight (TOF) analysis. The XICs for L-cysteine and cysteine-aldimine/quinonoid/thiazolidine were analyzed at positive ion TOF with mass of 122.0166 ± 0.0029 Da and 351.010 ± 0.006 Da, respectively. The XICs for aldimine and pyruvate were analyzed at negative ion TOF with mass from 314.75 to 315.25 Da and from 86.75 to 87.25 Da, respectively. Data is presented by plotting the intensity of the signal versus retention time for indicated products (black text) obtained from the reactants given (blue or red text) from LC-MS/MS analysis of the chemical reactions of L-cysteine, Fe^{3+} and PLP at 37°C (**F, G, H, I, J, M**) or 4°C (**K, L, M**). Blue italic text indicates reactions and conditions favorable for H_2S production while red indicates conditions not conducive for H_2S production. Numeric values are given in **Figure 5F.**

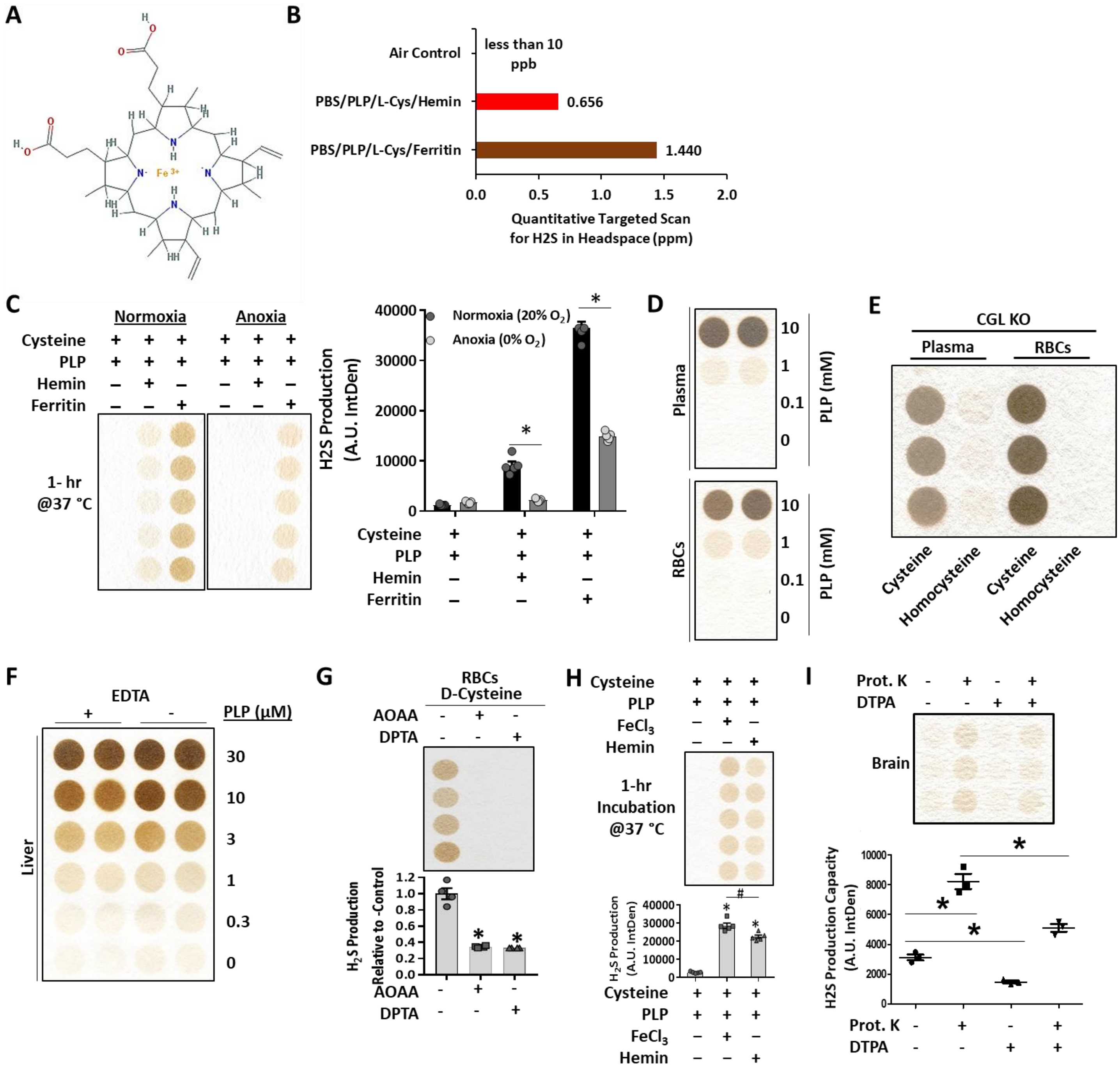

Supplementary Figure 6: Non-Enzymatic Production of H_2S in the Circulation and the brain. (**A**) Chemical structure of hemin. (**B**) Headspace H_2S detected in the selected ion flow tube mass spectrometry (SIFT-MS) with quantitative targeted scan for H_2S; n = 1/group. **(C)** H_2S production under conditions of normoxia (20% O_2) versus anoxia (0% O_2) in reaction mixture of cysteine, PLP and bound iron forms (hemin and ferritin); n = 5/group. Asterisk indicates the significance of the difference between two indicated groups; *P < 0.05. (**D**) PLP dose-dependently increases H_2S production from plasma and RBCs with the addition of L-cysteine as substrate; n = 2/group. (**E**) Cysteine, but not homocysteine, serves as a rapidly hydrolyzed substrate for H_2S production in plasma and RBCs; n = 3/group. (**F**) PLP dose-dependently increases enzymatic H_2S production in the liver, in which EDTA has minimal effect on this enzymatic production; n = 6/group. (**G**) H_2S production from RBCs using D-cysteine as substrate and ± inhibitors AOAA and PAG; n = 4/group. Asterisk indicates the significance of the difference versus the control group; *P < 0.05. (**H**) H_2S production catalyzed by free iron $FeCl_3$ versus bound iron hemin in reaction mixture of cysteine and PLP; n = 5/group. Asterisk indicates the significance of the difference versus the "L-cysteine and PLP" control and number sign indicates the significance of the difference between indicated groups; *,#P < 0.05. (**I**) H_2S production from the brain of 6-month old mice in reaction mixture of L-cysteine and PLP with ± pretreatment of DTPA and Prot. K; n = 3/group. Asterisk indicates the significance of the difference versus the brain protein sample with no DTPA or Prot. K pretreatment; *P < 0.05. All data were presented as mean ± SEM.

Supplementary Tables

Supplementary Table 1: Relevant components and their concentrations in DMEM and DMEM/F12 culture media.

Components	DMEM (mg L^{-1})	DMEM/F12 (mg L^{-1})
**Cysteine, Cystine, and VitB$_6$ (pyridoxine)**		
L-cysteine·HCl·H_2O	N.A.	17.56
L-cystine·2HCl	62.57	31.29
Pyridoxine·HCl	4.00	2.03
**Metal Ions**		
$Fe(NO_3)_3 \cdot 9H_2O$	0.1	0.05
$FeSO_4 \cdot 7H_2O$	N.A.	0.420
$ZnSO_4 \cdot 7H_2O$	N.A.	0.430
$CuSO_4 \cdot 5H_2O$	N.A.	9.091E-04
$CaCl_2$	200	116.000
$MgSO_4$	97.7	48.850

N.A.: Not available.
