# Supplementary material for: Non-enzymatic hydrogen sulfide production from cysteine in blood is catalyzed by iron and vitamin B6
Source: Commun Biol. 2019 May 21;2:194. doi: 10.1038/s42003-019-0431-5 (PMC6529520; doi:10.1038/s42003-019-0431-5)
Supplement: Supplementary file 2 — Reporting Summary [file 42003_2019_431_MOESM2_ESM.pdf]

nature research

Corresponding author(s): Christopher Hine , PhD. Assistant Staff/ Assistant Professor, Department of Cardiovascular and Metabolic Sciences, Cleveland Clinic Lerner Research Institute, Cleveland, Ohio.

Reporting Summary

Nature Research wishes to improve the reproducibility of the work that we publish. This form provides structure for consistency and transparency in reporting. For further information on Nature Research policies, see Authors & Referees and the Editorial Policy Checklist.

Statistical parameters

When statistical analyses are reported, confirm that the following items are present in the relevant location (e.g. figure legend, table legend, main text, or Methods section).

n/a	Confirmed	
☐	☒	The exact sample size (*n*) for each experimental group/condition, given as a discrete number and unit of measurement
☐	☒	An indication of whether measurements were taken from distinct samples or whether the same sample was measured repeatedly
☐	☒	The statistical test(s) used AND whether they are one- or two-sided<br>*Only common tests should be described solely by name; describe more complex techniques in the Methods section.*
☒	☐	A description of all covariates tested
☐	☒	A description of any assumptions or corrections, such as tests of normality and adjustment for multiple comparisons
☐	☒	A full description of the statistics including central tendency (e.g. means) or other basic estimates (e.g. regression coefficient) AND variation (e.g. standard deviation) or associated estimates of uncertainty (e.g. confidence intervals)
☐	☒	For null hypothesis testing, the test statistic (e.g. *F*, *t*, *r*) with confidence intervals, effect sizes, degrees of freedom and *P* value noted<br>*Give P values as exact values whenever suitable.*
☒	☐	For Bayesian analysis, information on the choice of priors and Markov chain Monte Carlo settings
☒	☐	For hierarchical and complex designs, identification of the appropriate level for tests and full reporting of outcomes
☒	☐	Estimates of effect sizes (e.g. Cohen's *d*, Pearson's *r*), indicating how they were calculated
☐	☒	Clearly defined error bars<br>*State explicitly what error bars represent (e.g. SD, SE, CI)*

Our web collection on statistics for biologists may be useful.

Software and code

Policy information about availability of computer code

Data collection	Data were collected using commonly used and/or commercially available software. Images of lead acetate/lead sulfide papers were scanned on an Epson J251A scanner using the Epson Scan software. UV/Vis and florescence data obtained from multimode plate readers was done on a SpectraMax i3x using the software SoftMax Pro 6.5.1. Metabolite analysis was performed on a Sciex 5600 TripleTOF and its associated software.
Data analysis	All Data analysis utilized commonly used and commercialy available software. Images of lead acetate/lead sulfide papers) were analyzed using the Integrated density (IntDen) feature on ImageJ software (version: Fiji Is Just ImageJ 2.0.0-rc-61/1.51n; Java 1.8.0_66 64-bit). Numerical data were analyzed using Microsoft Excel (Professional Plus 2013) and Graphpad Prism version 5.02.

For manuscripts utilizing custom algorithms or software that are central to the research but not yet described in published literature, software must be made available to editors/reviewers upon request. We strongly encourage code deposition in a community repository (e.g. GitHub). See the Nature Research guidelines for submitting code & software for further information.

Data

Policy information about availability of data

All manuscripts must include a data availability statement. This statement should provide the following information, where applicable:

- Accession codes, unique identifiers, or web links for publicly available datasets
- A list of figures that have associated raw data
- A description of any restrictions on data availability

The authors declare that the majority of the data supporting the findings of this study are available within the paper and its supplementary information files. Additionally, the raw data are available from the corresponding author upon reasonable request.

Field-specific reporting

Please select the best fit for your research. If you are not sure, read the appropriate sections before making your selection.

☒ Life sciences ☐ Behavioural & social sciences ☐ Ecological, evolutionary & environmental sciences

For a reference copy of the document with all sections, see nature.com/authors/policies/ReportingSummary-flat.pdf

Life sciences study design

All studies must disclose on these points even when the disclosure is negative.

Sample size	Determination of sample sizes of the final data presented were not established using pre-experimental calculations, but were instead established empirically based on preliminary experiments showing strong visual differences between groups that running N=3-6 per group was sufficient to reach statistical significance for the final experiments.
Data exclusions	No data were excluded from the analysis
Replication	All of the pertinent experiments and data presented in this manuscript were performed with at least N=3 per experiment and most experiments were run in duplicate independently by the first author and the corresponding (last) author. In these cases, we were able to replicate the outcomes of these experiments.
Randomization	Experimental randomization of animals was not necessary as there were no actual experimental variables/treatments associated with the animals. All animals were housed, fed, and sacrificed under identical conditions. Animals were used to obtain tissue and blood samples for downstream chemical and protein analysis under standard conditions and not under experimental conditions. However, choosing which mice out of the other mice in their cage/litter to sacrifice for these experiments was done randomly, in that there was no reason why one mouse was chosen over another mouse.
Blinding	Investigators were not blinded to group allocation during experimental setup or data collection and analysis, as blinding was not necessary due to there being no actual experimentation on animals, just tissue collection of mice under standard conditions. Chemical analyses assays were not performed blinded as this was not possible as the experimenter would have to know what samples and chemicals are being loaded in order to carry out the experiment. However, the operator of the SIFT-MS machine was blinded to the identify of the sample that was injected into the machine until after the data was obtained. All raw data relevant to these experiments are presented in the figures of this paper, so even though analysis of the data was not done blinded, the raw data is there for inspection.

Reporting for specific materials, systems and methods

Materials & experimental systems

n/a	Involved in the study	
☒	☐	Unique biological materials
☐	☒	Antibodies
☐	☒	Eukaryotic cell lines
☒	☐	Palaeontology
☐	☒	Animals and other organisms
☒	☐	Human research participants

Methods

n/a	Involved in the study	
☒	☐	ChIP-seq
☒	☐	Flow cytometry
☒	☐	MRI-based neuroimaging

Antibodies

Antibodies used	Primary Antibodies: Anti-MPST (Abcam ab85211, lot GR31068-12), anti-Cystathionase (Abcam ab151769, lot GR311382-7), Anti-CBS (Abcam ab135626, lot GR262599-4), Anti-alpha Tubulin (Abcam ab4074, lot GR3194994-1).
Validation	All antibodies used in this manuscript were purchased directly from Abcam, and thus underwent their stringent production and testing procedures. Additionally, I have published previous work (Hine, et al. Cell 2015 and Hine, et al. Cell Metabolism 2017) validating the Cystathionase and CBS antibodies. The alpha Tubulin antibody has been published in at least 154 publications. The MPST antibody was validated by blots presented on Abcam's webpage as well as data in this manuscript, showing strong MPST expression in mouse brain, which has been published previously.

Eukaryotic cell lines

Policy information about cell lines

Cell line source(s)	Cell Line: NCTC 1469 (mouse liver epithelial, normal) purchased directly from American Type Culture Collection (ATCC) (catalog number ATCC CCL-9.1) just prior to the start of these experiments
Authentication	We did not authenticate this cell line as it was purchased directly from ATCC, and expanded and grown in an incubator that did not house any other type of cell during this experimental time.
Mycoplasma contamination	Cell lines were not tested for mycoplasma contamination as they were purchased directly from the vendor American Type Culture Collection (ATCC), expanded for only several population doublings, frozen and then aliquots used only before the culture reached 20 passages.
Commonly misidentified lines (See ICLAC register)	none were used

Animals and other organisms

Policy information about studies involving animals; ARRIVE guidelines recommended for reporting animal research

Laboratory animals	Animals: Mice (Mus musculus). Strains: C57BL/6J (Female, 6 months of age), and Cystathionine Gamma-Lyase wildtype and knockout mice on C57BL/6J × 129SvEv background (male, 6 weeks old)
Wild animals	Study did not involve wild animals
Field-collected samples	Study did not involve samples collected from the field
